# Antibody-based drug delivery systems for cancer therapy: Mechanisms, challenges, and prospects

**DOI:** 10.7150/thno.72594

**Published:** 2022-05-01

**Authors:** Zhoujiang Chen, Ranjith Kumar Kankala, Zhiyong Yang, Wei Li, Songzhi Xie, Hanmei Li, Ai-Zheng Chen, Liang Zou

**Affiliations:** 1 School of Food and Bioengineering, Institute for advanced study, Chengdu University, Chengdu 610106, Sichuan, PR China.; 2 Institute of Biomaterials and Tissue Engineering, Huaqiao University, Xiamen 361021, Fujian, PR China.; 3 Department of Clinical Pharmacy, Affiliated Hospital of Chengdu University, Chengdu University, Chengdu 610081, Sichuan, PR China.; 4 School of Basic Medical Sciences, Chengdu University, Chengdu 610106, Sichuan, PR China.

**Keywords:** Antibody-based therapy, Immunotherapy, Drug delivery system, Controlled release

## Abstract

In recent years, antibody-based cancer therapy has emerged as one of the efficient therapeutic strategies, such as immune checkpoint inhibitors (ICIs), angiogenesis inhibitors, antibody-drug conjugates (ADCs), multi-specific antibodies, and chimeric antigen receptor T (CAR-T) cells, among others. To date, various drug delivery platforms have been developed to improve the bioavailability, delivery convenience, and reduced toxicity towards increased therapeutic efficacy of antibodies. Herein, we emphasize the clinical manifestations of various antibody-based tumor therapies, highlighting their mechanisms and applications for cancer therapy. Further, based on the problems to be solved in the current clinical application of antibodies, and combined with the advanced drug delivery technologies, we discuss the roles of antibody-based drug delivery systems (DDSs) in cancer therapy, such as enhanced patient compliance and regulating the tumor microenvironment for combined therapy. By expounding the importance of DDSs and discussing the challenges and prospects of their implementation, we suggest that pharmaceutical enterprises and scientists develop appropriate antibody-based delivery platforms.

## Introduction

Indeed, cancer is one of the major healthcare challenges, accounting for millions of deaths globally. Despite the success in developing various therapeutic strategies, there are several deficiencies in traditional chemotherapy, including limited curative efficacy, easy drug resistance, recurrence, and side effects. In recent times, antibodies have emerged as one of the predominant therapeutic options for various ailments such as cancer, migraine headaches, infection, asthma, arthritis, Crohn's disease, transplant rejection, psoriasis, and hereditary angioedema, among others 1. In 1975, monoclonal antibodies (mAbs) were obtained on a large scale by the hybridoma technique, showing their great potential for diverse clinical applications 2. Since Muromonab-CD3 (Orthoclone OKT3, a murine-derived mAbs) was approved first clinically in 1986, more than 70 mAbs therapeutics have been approved and available in the market. At least 570 mAbs have been researched in clinical trials worldwide by 2020 1. Despite the success, these murine-derived mAbs (for instance, Orthoclone OKT3) are recognized as foreign antigens by the human immune system resulting in some adverse events, such as immunogenicity, substantially hindering their development and translation 3. Thus, humanized therapeutic antibodies (such as daclizumab) and fully human antibodies (such as adalimumab) have been developed and approved 4, 5. Furthermore, PEGylation of therapeutic antibodies showed an improved half-life and reduced immunogenicity, such as certolizumab pegol (Cimzia®), approved to treat multiple chronic inflammatory conditions in 2008 6. In addition, antibody-drug conjugates (ADCs) and bispecific antibodies (BsAbs) are emerging classes of antibody-based drugs, which show high potential for the treatment of cancer 7, 8.

Till 2021, more than 40 antibodies have been approved by the United States Food and Drug Administration (US-FDA) for cancer treatment. Although these antibody-based therapies have become the most significant biotherapeutics, these innovative therapeutic agents still suffer from major shortcomings, for instance, challenges in crossing various complex biological barriers in the body. Firstly, as a kind of protein, these antibodies are susceptible to enzymatic and chemical constituents in the physiological environment. Some antibody fragments are also rapidly metabolized in the body. For instance, the half-life of blinatumomab is only about 2 hours, leading to prolonged intravenous infusion 9. Secondly, antibodies often cause off-target cytotoxicity and adverse events, such as cytokine release syndrome (CRS) and organ toxicity. Some adverse reactions even endanger patients' lives, limiting the comprehensive application of antibody therapy to a certain extent. Thirdly, the response rate of the indications of most antibody-based immunotherapies is usually low due to cancers showing primary, adaptive, and acquired drug resistance mechanisms 10. In general, the objective response rate of programmed death 1 (PD-1) immune checkpoint inhibitors (ICIs) to unstable microsatellite cancers is about 35% 11. In addition, for some solid tumors, the immunosuppressive effect of “cold” tumors seriously affects the efficacy of immunotherapy 12. Fourthly, patient compliance is clinically required to execute the anticipated therapeutic events. For example, a continuous infusion cycle of 28 days is required to treat acute lymphoblastic leukemia (ALL) with blinatumomab, requiring sophisticated administration equipment that affects the movement and mobility of patients 13. Based on these considerations, it is essential to currently solve these challenges, which could be possible by employing various delivery systems effectively.

Indeed, the nanomedicine-based therapy is intended to alter the pharmacokinetics (PK), reduce the toxicity of traditional chemotherapeutics, and promote the drug accumulation precisely in the tumor tissue. Many drug-based formulations have been approved for cancer treatment, such as liposomes, human serum albumin, micelles, and many others undergoing Phase 2/3 clinical trials 14. These drug delivery systems (DDSs) play important roles in improving the current therapeutic regimens by optimizing the therapeutic efficacy and PK standpoints. As a key class of emerging and rapid development drugs, antibody-based biotherapeutics can provoke antitumor immune response or target tumor cells. However, they still face similar challenges as chemotherapy, requiring more attention to improve their delivery patterns than chemotherapy chemotherapeutics. In this regard, the controlled release of antibodies by DDS has a broad prospect to overcome the challenges, such as sustained release for medication convenience, augmented efficacy, and reduced toxicity. However, the large molecular weight and biological activity of antibodies are considerably different from small-molecule chemotherapeutic drugs, making it more difficult to control the release of antibodies. In any case, the fabrication of a DDS is very conducive to applying antibody-based therapies to be clinically efficient.

Considering these attributes, we emphasize the mechanisms and applications of mAbs and their derivatives, including ICIs, Fc-mediated effectors, angiogenesis inhibitors, receptor signal blockers, ADCs, multi-specific antibodies, and chimeric antigen receptor T (CAR-T) cells. Further, we summarize the problems associated with the biological barriers that these bio-therapeutics face in clinics. We aim to analyze the application potential and challenges of various delivery systems for antibody-based therapy. To the best of our knowledge, exceptional delivery systems have been developed for the clinical application of mAbs. Based on these aspects, we then present the advanced DDSs, that are aimed at increasing drug convenience, reducing toxicity, and increasing efficiency for antibody therapy, highlighting their clinical transformation potential.

## Anticancer mechanisms and clinical applications of mAbs

**Figure 1:**
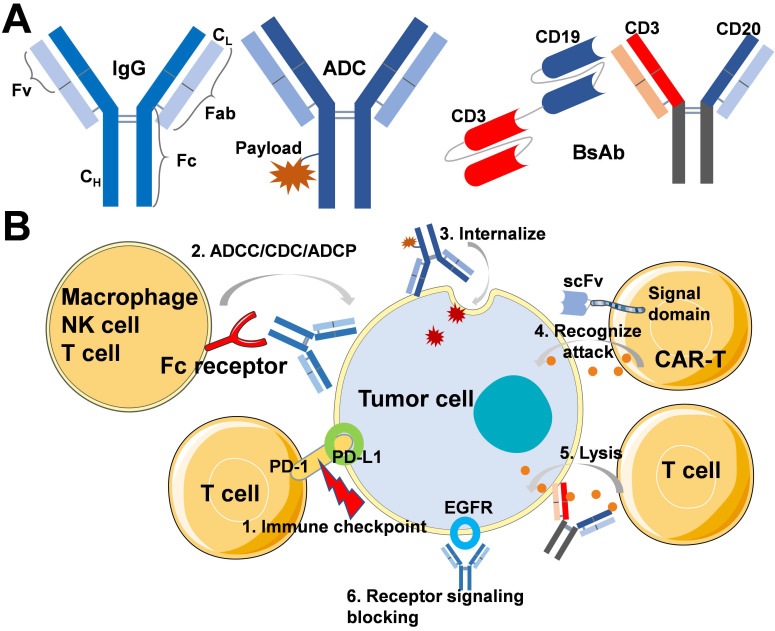
** (A)** Schematic of IgG, ADC, BsAbs without Fc region, such as blinatumomab and BsAbs containing Fc region, such as mosunetuzumab. **(B)** Mechanisms of action for the treatment of tumor: 1. Immune checkpoint inhibitors block the effects of inhibitory pathways and augment the host immune system for recognition and elimination of tumor cells; 2. mAb specifically targets cancer cells by binding the overexpressed surface receptor, and the Fc region-mediated ADCC, CDC, and ADCP leads to cytolysis or phagocytosis by nature killing the cell, cytotoxic T lymphocytes, and macrophage; 3. ADC internalizes mediated with the receptor, and released cytotoxin causes cancer cell death; 4. CAR-T cell recognizes and attacks cancer cells; 5. BsAbs recruitment of T cells to cancers through binding of tumor-cell-surface antigens to immune cells, then release granzymes and perforin to kill cancer cells; 6. The binding of inhibitors to EGFR can prevent ligand-induced receptor autophosphorylation and the activation of receptor-related kinases to inhibit cell growth, reduce pro-inflammatory cytokines and vascular growth factor production.

In general, mAbs immunoglobulins (Ig) are classified into 5 sub-types, such as IgA, IgD, IgE, IgG, and IgM. Among all, IgG (Mol. Wt. of 150 kDa) is the most relevant therapeutic antibody currently approved for disease treatment. The molecular structure of IgG possesses two separate identical heavy chains (C_H_) and light chains (C_L_) (**Figure 1A**). In addition, IgG is broadly classified into the Fab and Fc regions, in which the Fab regions of different IgG bind to a different specific epitope on antigens. At the same time, Fc is essential for antibody-dependent cell-mediated cytotoxicity (ADCC), antibody-dependent cellular phagocytosis (ADCP), and complement-dependent cytotoxicity (CDC) 15. Moreover, as shown in **Figure 1A**, structures, such as ADCs and BsAbs, are derived based on conventional mAbs. Natural properties of mAbs molecules include high affinity and specificity to antigens, which then block receptor-ligand interactions and activate the immune system by CDC, ADCC, and ADCP. According to the various mechanisms of action of antibodies, on the one hand, some antibodies can activate the immune system to recognize and eliminate cancer cells, even preventing the occurrence of cancer. On the other hand, some antibodies can specifically bind with the tumor-associated antigens (TAAs), which are overexpressed in malignant cells but not expressed or only expressed at a low level in normal cells, providing opportunities for targeted chemotherapy. **Figure 1B** shows the mechanisms of action about ICIs, mAbs for Fc-mediated effector, ADCs, CAR-T cells, BsAbs, and receptor signaling blocking inhibitors. In this section, we discuss different kinds of antibody-based anticancer biotherapeutics, including the mechanism of action and clinical application.

### Immune checkpoint inhibitors (ICIs)

In 2011, ipilimumab as an immune checkpoint inhibitor was approved by US-FDA, which introduced a new therapeutic mechanism for cancer treatment 16. Immune checkpoints certainly influence the pathways, affecting the efficacy of immune cells. Malignant cells promote an immunosuppressive nature that favors immune evasion and cancer growth while generating a highly immunosuppressive tumor microenvironment (TME) 17. Under such circumstances, ICIs can block the effects of inhibitory pathways, which augment the host immune system to recognize and eliminate tumor cells. Various commercial ICIs approved so far, including inhibitory immune checkpoints of cytotoxic T lymphocyte-associated molecule-4 (CTLA-4, also known as CD152), PD-1, and programmed cell death receptor-1 ligand (PD-L1), are summarized in **Table 1**. Furthermore, the next generation of immune checkpoint inhibitors for new targets (such as CD223, T-cell immunoglobulin mucin 3) is under ongoing clinical trials 18.

Indeed, T cells are activated by T cell receptors (TCR), but CTLA-4 is upregulated on the surface of active T cells to prevent the stimulation of TCR 19. During the activation of T cells, the CD28 receptor on the surface of T cells binds to the B7 ligand on antigen-presenting cells (APCs), providing a necessary secondary activation signal for T cell activation. However, the higher affinity of CTLA-4 receptor to B7 ligand makes CD28 receptor unable to bind to the ligand, resulting in ineffective T cells 20. Thus, anti-CTLA-4 antibodies could block CTLA-4 and enhance effector T-cell activation and proliferation. Ipilimumab (MDX-010, Yervoy ®), an anti-CTLA-4 mAb, can block the molecules and lead to immune-mediated anticancer response for melanoma, renal cell carcinoma (RCC), colorectal cancer, malignant pleural mesothelioma, hepatocellular carcinoma (HCC), and non-small cell lung cancer (NSCLC) 21. Ipilimumab injection is administered intravenously in doses of 1 to 10 mg/kg, depending on different indications. Across the clinical trials, severe infusion-related reactions, such as chills, dizziness, fever, itching, and wheezing, occurred in 2.9%-12%, so it is necessary to interrupt the administration or slow down the infusion rate to moderate the reactions. Ipilimumab is a recombinant IgG1 immunoglobulin, and the molecular weight of ipilimumab is about 148 kDa. According to the manufacturer's exploration, the antibody showed good pharmacokinetic characteristics, with a mean terminal half-life of 15.4 days and mean clearance was 16.8 mL/h. Though ipilimumab showed better tolerated than chemotherapy, the immune-mediated adverse reactions (IMAR) associated with ipilimumab are unique and usually need to be treated with steroids. Severe and fatal IMAR can occur in various organ systems and tissues during treatment or after discontinuation, including colitis, hepatitis, endocrinopathies, adverse dermatologic reactions, and pneumonitis.

PD-1 is an immunosuppressive costimulatory signal receptor, expressed on activated T cells and B cells. Often, PD-1 binds to its ligands such as PD-L1 and PD-L2, leading to the transmission of a negative costimulatory signal, limiting the activation of T cells, and blocking T cells from recognizing and destroying cancer cells. Indeed, PD-L1 can even induce rapid tumor growth, and it is commonly upregulated in cancer cells and expressed dendritic cells, lungs, blood vessels, and placenta 22. In the process of tumor development, the combination of PD-1 and PD-L1 will cause tumor immune escape, mainly by inhibiting the activity of tumor-infiltrating lymphocytes, inhibiting the production of cytotoxic T lymphocyte perforin and granular enzymes, reducing the secretion of anti-inflammatory factors, arresting T cell cycle, as well as promoting tumor cell epithelization and tumor infiltration 23. Thus, PD-1 and PD-L1 blocking antibodies can relieve the immunosuppression and achieve anti-cancer effect. As depicted in **Table 1**, several PD-1/PD-L1 blocking antibodies have already been approved to treat multiple hematologic and solid malignancies. Several anti-PD-1 agents, for instance, nivolumab, pembrolizumab, and cemiplimab are prescribed to treat melanoma, NSCLC, RCC, classical Hodgkin lymphoma (cHL), urothelial carcinoma, colorectal cancer, and HCC, among others 23. Atezolizumab, avelumab, and durvalumab are PD-L1 blocking antibodies indicated for Merkel cell carcinoma (MCC), urothelial carcinoma (UC), RCC, NSCLC, and so on. Hence, the indications for PD-1 blocking antibodies are broader than for PD-L1 blocking antibodies. Generally, PD-1 and PD-L1 blocking antibodies are prepared as solution injections for intravenous administration. The geometric mean elimination half-life of these antibodies is usually several days to more than 20 days. However, it is required to pay attention to the autoimmune toxicities and infusion-related reactions, which are similar to anti-CTLA-4 antibodies. In general, antibodies targeting PD-1 result in a more serious and higher incidence of adverse reactions than anti-PD-L1 antibodies 24. The immune toxicity induced by ICIs mainly presents the following common characteristics 25. First, it does not involve multiple organs of the patient simultaneously. Second, the onset of toxicity is delayed regularly. Third, there is no apparent correlation between toxicity and dose, and reducing dose can provoke their recurrence.

In addition to the three immune checkpoint inhibitors mentioned above, several agents for new inhibitory targets are currently undergoing clinical trials and evaluation, such as T-cell immunoglobulin and mucin-domain domain-containing molecule-3 (TIM-3), and lymphocyte-activation gene 3 (LAG-3), among others 18. TIM-3 is a receptor expressed on the surface of T cells, B cells, natural killer (NK) cells, macrophages, and even cancer cells. It causes T-cell depletion and stimulates tumor growth, such that blocking of TIM-3 reduces immunosuppression and promotes T-cell proliferation. Sabatolimab, a humanized IgG4 anti-TIM-3 antibody, was developed by Novartis to treat acute myeloid leukemia (AML) and myelodysplastic syndrome (MDS). Based on the results of the concurrent Phase-2 and Phase-3 clinical trials, sabatolimab for the treatment of MDS is expected to be filed in 2022-23 26. In addition, Sym023 and TSR-022 are anti-TIM-3 antibodies developed for advanced solid tumors and lymphomas 18. Anti-TIM-3 agents have garnered enormous attention due to their wide expression and synergistic effects with other ICIs and chemotherapy drugs. However, in the case of combined administration, concerns regarding adverse reactions such as infection and fatigue are inevitable. LAG-3 (also known as CD223), an immune checkpoint like PD-1, is expressed by activated T cells, NK cells, B cells, and dendritic cells. LAG-3 blockade prevents immune failure and inhibits tumor growth, and may enhance the effect of other ICIs. Relatlimab is a human IgG4 anti-LAG-3 antibody that has been tested in clinical trials in combination with nivolumab (an anti-PD-1 antibody) to treat unresectable or metastatic melanoma 27. Similar to sabatolimab, it is essential to determine the combined dose with anti-LAG-3 antibodies and focus on enhancing adverse reactions while used in combination.

In a case, the clinical efficacy showed that the effective rate of PD-1/PD-L1 blocking antibodies was about 80% in lymphoma, about 60% in high microsatellite instability (MSI) tumors, and fluctuated between 10% and 30% in other common solid tumors 28. Thus, despite the success of anti-CTLA-4 and anti-PD-1/PD-L1 therapies, combination therapy often attempts to improve the response to treatment. For example, on the one hand, anti-CTLA-4 antibodies can promote the early activation of T cells in lymphoid tissue. On the other hand, anti-PD-1 antibodies mainly inhibit T cell failure in tumor tissues 29. In general, the difference in the efficacy of ICIs to patients is mainly due to the different expression of biomarkers, tumor mutation burden (TMB), and MSI 30. As shown in **Table 1**, commercial ICIs are currently administered primarily by intravenous infusion and subcutaneous dosage forms, such as envafolimab (a humanized single-domain anti-PD-L1 antibody) 31.

### mAbs for Fc-mediated effector

Initially, the development of mAbs as anticancer drugs is mainly based on the binding of Fab to antigen, while Fc can be used to activate many immunological pathways through interactions with Fcγ receptors 32. In this way, the Fcγ receptor on immune cells binds to Fc, induces, and activates effector cells, phagocytes, complement, leading to the mechanisms of ADCC, ADCP, and CDC to kill tumor cells. After the antibody binds to the target antigen, Fc recruits Fcγ receptor-expressing immune cells, including T cells, NK cells, and macrophages, and then induces the release of perforin and granzyme, or phagocytosis to clear tumor cells. In addition, after the binding of Fc and the C1q component, the complement system is activated to produce the membrane attack complex and destroy the tumor cell membrane 33. As seen in **Table 2**, many commercial antibody-based drugs have been developed based on these mechanisms. The targeted antigens of these mAbs include CD19 (tafasitamab), CD20 (ofatumumab, obinutuzumab, and rituximab), CD38 (daratumumab, and isatuximab), CD52 (alemtuzumab), GD-2 (naxitamab, and dinutuximab), epidermal growth factor receptor (EGFR, necitumumab), signaling lymphocytic activation molecule family (SLAMF7, elotuzumab), CC chemokine receptor type 4 (CCR4, mogamulizumab), and human epidermal growth factor receptor 2 (HER2, trastuzumab, margetuximab, and pertuzumab).

CD19 antigen is expressed on the surface of pre-B and mature B lymphocytes and several B-cell malignancies but not expressed on other normal cells. Upon binding to CD19, tafasitamab induces B-cell lysis through apoptosis and immune effector mechanisms (ADCC and ADCP) 34. In combination with the chemotherapeutic drug (lenalidomide), tafasitamab is approved to treat relapsed or refractory diffuse large B-cell lymphoma (DLBCL). The terminal elimination half-life of tafasitamab is approximately 17 days, and over several days of treatment, tafasitamab rapidly acts by reducing the peripheral blood B cell counts. The main adverse reactions of tafasitamab are bone marrow suppression, including anemia, neutropenia, and thrombocytopenia. Similar to CD19, CD20 antigens are also expressed on pre-B- to mature B-lymphocytes. The typical antibodies that target CD20 include ofatumumab, obinutuzumab, and rituximab used to treat chronic lymphocytic leukemia (CLL) and non-Hodgkin's lymphoma (NHL) 35. The predominant mechanisms of action are through CDC, ADCP, and ADCC-mediated B cell ablation, and even directly activate intracellular death signaling pathways. However, it should be noted that these CD20-directed cytolytic antibodies can cause hepatitis B virus (HBV) reactivation and lead to fulminant hepatitis, hepatic failure, and even death 36. In addition, CD52 is another antigen present on the surface of B and T lymphocytes, and some bone marrow cells also express certain levels of CD52 37. Alemtuzumab is an anti-CD52 mAb, which is considered a single agent for treating B-cell chronic lymphocytic leukemia (B-CLL) by antibody-dependent cell-mediated lysis. GD2, a disialoganglioside overexpressed on neuroblastoma and GD2-binding monoclonal antibodies (naxitamab and dinutuximab), is prescribed for the treatment of children with high-risk neuroblastoma 38. To this end, the lymphocytic signaling activation molecule family member 7 (SLAMF7) is mostly expressed on myeloma, NK cells, and plasma cells 39. Elotuzumab directly activates NK cells through both the SLAMF7 pathway and ADCC for the treatment of multiple myeloma (MM). Hepatotoxicity is a significant clinical adverse reaction by elotuzumab treatment, requiring monitoring of liver enzymes regularly.

CD38 is a transmembrane glycoprotein mainly expressed on plasma cell neoplasms, for instance, MM 40. Isatuximab and daratumumab, CD38-directed cytolytic antibodies, have been used for the treatment of adult patients with MM through inducing apoptosis of cancer cells and activating immune-effecting mechanisms including ADCC, ADCP, and CDC. The most common adverse reactions of CD38-directed cytolytic antibodies are neutropenia, pneumonia, upper respiratory tract infection, and diarrhea. Daratumumab comes in two administration forms, Darzalex^®^ for intravenous administration and Darzalex Faspro^®^ for subcutaneous use. Until now, few anti-tumor antibodies have been administered subcutaneously to reduce infusion-related reactions and increase the convenience of medication. Similar to Darzalex Faspro^®^, Herceptin Hylecta® is also a combination of trastuzumab and hyaluronidase. Hyaluronidase is added in the preparation to increase the permeability of the subcutaneous tissue by depolymerizing hyaluronan 41. Trastuzumab can selectively bind with high affinity to human epidermal growth factor receptor 2 (HER2). Hyaluronidase can increase the dispersion and absorption of co-administered antibodies during subcutaneous administration. Upon binding to HER2-expressing tumor cells, trastuzumab, margetuximab, and pertuzumab inhibit tumor cell proliferation and mediate cell death by ADCC. They are indicated for the treatment of metastatic HER2-positive breast and gastric cancer. Due to the adverse reactions of left ventricular dysfunction, the cardiac function needs to be evaluated before and during treatment.

### Angiogenesis inhibitors and receptor signaling blocking

In general, solid tumors often show some characteristics, such as irregular vascularization, abnormal tumor immune microenvironment, and hypoxia. Therefore, inhibiting tumor angiogenesis by targeting angiogenic factors has become a way to treat tumors 42. **Table 3** shows the commercialized mAbs involved in angiogenesis inhibition and receptor signaling blocking. Bevacizumab inhibits angiogenesis by binding vascular endothelial growth factor (VEGF) and blocking the interaction between VEGF and its receptor. Administration of bevacizumab can reduce microvascular growth and inhibit the progression of metastatic colorectal cancer, NSCLC, RCC, and recurrent glioblastoma. Ramucirumab, a vascular endothelial growth factor receptor 2 (VEGFR2) antagonist, inhibits ligand-induced proliferation and migration of human endothelial cells towards the treatment of advanced or metastatic gastric and lung cancer with disease progression in combination with chemotherapy.

The EGFR is a transmembrane glycoprotein that is overexpressed in the colon and rectal cancers 43. Interaction of EGFR with EGF leads to phosphorylation and activation of related intracellular proteins, which in turn regulate the transcription of genes related to cell growth, survival, motility, and proliferation. Panitumumab is an EGFR antagonist which binds specifically to EGFR on tumor cells and then competitively inhibits the binding of ligands for EGFR. The binding of panitumumab to EGFR can prevent ligand-induced receptor autophosphorylation and the activation of receptor-related kinases to inhibit cell growth and reduce pro-inflammatory cytokines and vascular growth factor production. Therefore, panitumumab is used to treat some subtypes of metastatic colorectal cancer. In addition to panitumumab, cetuximab also specifically targets EGFR to treat squamous cell carcinoma of the head and neck. Dermatologic and soft tissue toxicity, ocular toxicities, and pulmonary fibrosis are the common adverse reactions of this antibody.

Olaratumab is a platelet-derived growth factor receptor alpha (PDGFR-α) blocking antibody, often prescribed for treating adult patients with soft tissue sarcoma 44. PDGFR-α is a receptor tyrosine kinase expressed on mesenchymal origin cells. This receptor on sarcomas and stromal cells plays a role in cell growth, chemotaxis, mesenchymal stem cell differentiation, and tumor metastasis. The interaction between olaratumab and PDGFR-α prevents binding of the receptor and downstream PDGFR-α pathway signaling.

### Antibody-drug conjugates

Chemotherapy using cytotoxic drugs has shown great success in the treatment of cancer. However, the off-target cytotoxicity often causes serious adverse reactions, affecting the quality of life of patients and even threatening the life of patients. Hence, highly cytotoxic agents conjugated with mAbs possess sufficient tumor antigen affinity and specificity 45. The cytotoxic payloads can be microtubule-disrupting agents and DNA damaging agents, which should be acceptable to destroy the tumor cells. The antibody is an important part of ADC design with high binding power affinity and tumor-associated antigens. Moreover, the antibody should be well-tolerated retention, low immunogenicity, low cross-reactivity, and appropriate binding properties. In addition, chemical linkers are utilized to connect the cytotoxic payload to the mAb and maintain the stability of the ADC. All US-FDA-approved ADCs use the amino group of lysine on the antibody or the sulfhydryl group reduced by the interchain disulfide bond to be coupled to the drug-linkers. In this way, the payload is grafted to specific sites of the antibody, and the appropriate drug‑to‑antibody ratio is set to get an excellent therapeutic index by optimizing antibody stability, toxicity, and pharmacokinetic half-life. After binding to antigens expressed on the surface of tumor cells, ADCs enter cells via receptor-mediated endocytosis and release payloads to kill the cells. It is worth noting that ADCs generally not only have the efficacy of chemotherapy but also activate the ADCC and ADCP mechanisms of immune effector cells 46. Different from others, zevalin® is a radiotherapeutic agent by conjugating CD20 target monoclonal antibody with Yttrium-90. As presented in **Table 4**, 11 ADCs with different targets have been approved for marketing, and their indications include hematologic and solid tumors. All ADCs are administered by intravenous infusion, such that the systemic toxicity of chemotherapy is often challenging. The most common adverse reactions include peripheral neuropathy, hematologic toxicities, and hepatotoxicity, among others.

### BsAbs and CAR-based therapies

BsAbs directly recognize two different antigens on tumor or immune cells. BsAbs are artificially designed to act as a synergistic mechanism or improve the therapy index compared to monospecific antibodies. BsAbs have been widely studied for use in cancer, hemophilia, and autoimmune diseases, among others 47. In treating tumors, they are generally designed as one antigen-binding site against a CD3 receptor on cytotoxic T lymphocytes and the other against the receptor on the surface of tumor cells. BsAbs recruitment of T cells to cancers and eventually induce the T cells to release granzymes and perforin to destroy cancer cells. Although more and more BsAbs and multi-specific antibodies are being developed and entered into clinical trials, only blinatumomab is commercially available. The first BsAb approved for cancer treatment was catumaxomab, which activates endogenous T cells by connecting CD3 with epithelial cell adhesion molecule (EpCAM) on cancer cells, indicating for malignant ascites therapy 48. Catumaxomab was only used for intraperitoneal injection because intravenous administration may cause severe toxicity at low doses, related to Fc-mediated off-target T cell activation in the liver 8. However, catumaxomab was withdrawn from the market in 2017 for commercial reasons. Different from catumaxomab, Blincyto® (blinatumomab) is a CD19- and CD3-targeting antibody fragment without Fc region, and the current indication is mainly for the treatment of adults and children with B-cell ALL (**Table 5**) 49. Hence, blinatumomab has a short half-life and must be continuously administered intravenously for a long time to maintain stable plasma concentrations. Long-term follow-up survival data showed that the overall survival (OS) of 8-10 years after treatment was more than 50%, but the neurological adverse events and CRS are common adverse reactions 48.

CAR-T targets and kills cancer cells by inserting genes expressing antibodies targeting tumor cells into T cells 1. It can be considered antibody-based therapy because it is based on antibody recognition of targeted antigens. CAR-T cells combine chimeric antigen receptors with transplanted T cells through genetic technology, including extracellular antibody fragment scFv and cytoplasmic signal domain. In this way, CAR-T cells can specifically recognize tumor surface antigen to cytotoxic T-cell engagement. For example, Breyanzi® (lisocabtagene maraleucel) is a CD19 targeting autologous CAR-T cell therapy, which contained an FMC63 mAb-derived scFv, an IgG4 hinge region, a CD28 transmembrane domain, a 4-1BB costimulatory domain, and CD3 zeta activation domain 50. After binding to CD19 overexpressed on tumor cells, it induces activation of CAR-T cells to release cytotoxic agents for the killing of cancer cells. CAR-T cell therapy offers an excellent clinical effect on hematological tumors. As shown in **Table 5**, all the CAR-T therapies on the market use autologous T cells. Autologous CAR-T cell therapies have a better safety profile than allogeneic ones, but this personalized treatment cannot be used on a large scale. Allogeneic CAR-T cells are difficult to survive *in vivo*, but they are more suitable for large-scale production. Therefore, CAR-T cell therapies still need to be improved (such as in manufacturing and storage), reduce costs and improve shelf-life 51. The main adverse effects of CD19-directed CAR-T treatment included CRS, and infection.

Moreover, the design of multi-directed CAR-T cells is rational because of the predisposition of CD19 immune escape following CD19-directed CAR-T therapy. It is concluded that CD19/CD22 CAR-T cell therapies often cause slight CRS toxicity and have a comparable complete response and progression-free survival rates than the CD19 CAR-T therapies in ALL patients 52. In addition, the persistence of CD19/CD22 CAR-T cells is reduced compared to CD19 CAR-T cells in pediatric ALL 53. Some preclinical data on CD19/CD20 CAR-T cells showed that it could prevent the CD19 immune escape by malignant B cells 54. NK cells are one of the major lymphocyte subsets that can kill cancer cells through the ADCC mechanism. Similar to CAR-T, chimeric antigen receptor natural killer (CAR-NK) therapy is another cellular immunotherapy for treating both hematological and solid tumors. The NK cells sources included peripheral blood, umbilical cord blood, hematopoietic progenitors from cord blood and human-induced pluripotent stem cells, and CAR designs based on different signaling pathways that activate NK Cells has also been developed 55. Although there at least 13 clinical trials related to CD19 CAR-NK therapy have been evidenced, there are no commercial products yet available in the market. NK cells are harder to expand and more resistant to genetic engineering, which becomes an obstacle for the manufacturing of CAR-NK cells. Fortunately, unlike allogenic T cells, NK cells do not produce graft-versus-host disease and have excellent potential as safety and “off-the-shelf” CAR-based immunotherapy.

### mAbs in late-stage clinical studies for cancer indications

The advantages of monoclonal antibodies in treating tumors have led to their rapid development. In recent years, many antibody drugs have entered Phase-II or Phase-III clinical trials 26. As shown in **Table 6**, multi-specific antibodies and ADCs have become the favorites of biopharmaceutical industries in recent years. For instance, mosunetuzumab is a BsAb developed by Roche that targets CD20 and CD3, directing T cells to eliminate malignant B cells and avoiding the destruction of participating T cells. Mosunetuzumab showed good efficacy for relapsed or refractory follicular lymphoma patients after subcutaneous or intravenous administration in a Phase 1/2 clinical trial. Its efficacy in combination with lenalidomide was investigated in Phase 3 clinical trial. Mirvetuximab soravtansine is an ADC developed by ImmunoGen, which is used to treat epithelial malignancies by coupling humanized folate receptor alpha antibody with DM4 *via* disulfide linker. In addition, radiolabeled (iodine-131) antibodies and some new immune checkpoint inhibitor antibodies have been developed. In recent years, many antibodies have been submitted for clinical evaluation in the United States, the European Union, China, Japan, and other countries. Although some antibodies show some deficiencies in clinical application, the total number of approved antibody drugs shows an increasing trend, presenting favorable commercialization prospects.

## Drug delivery systems for antibody-based therapy

**Figure 2:**
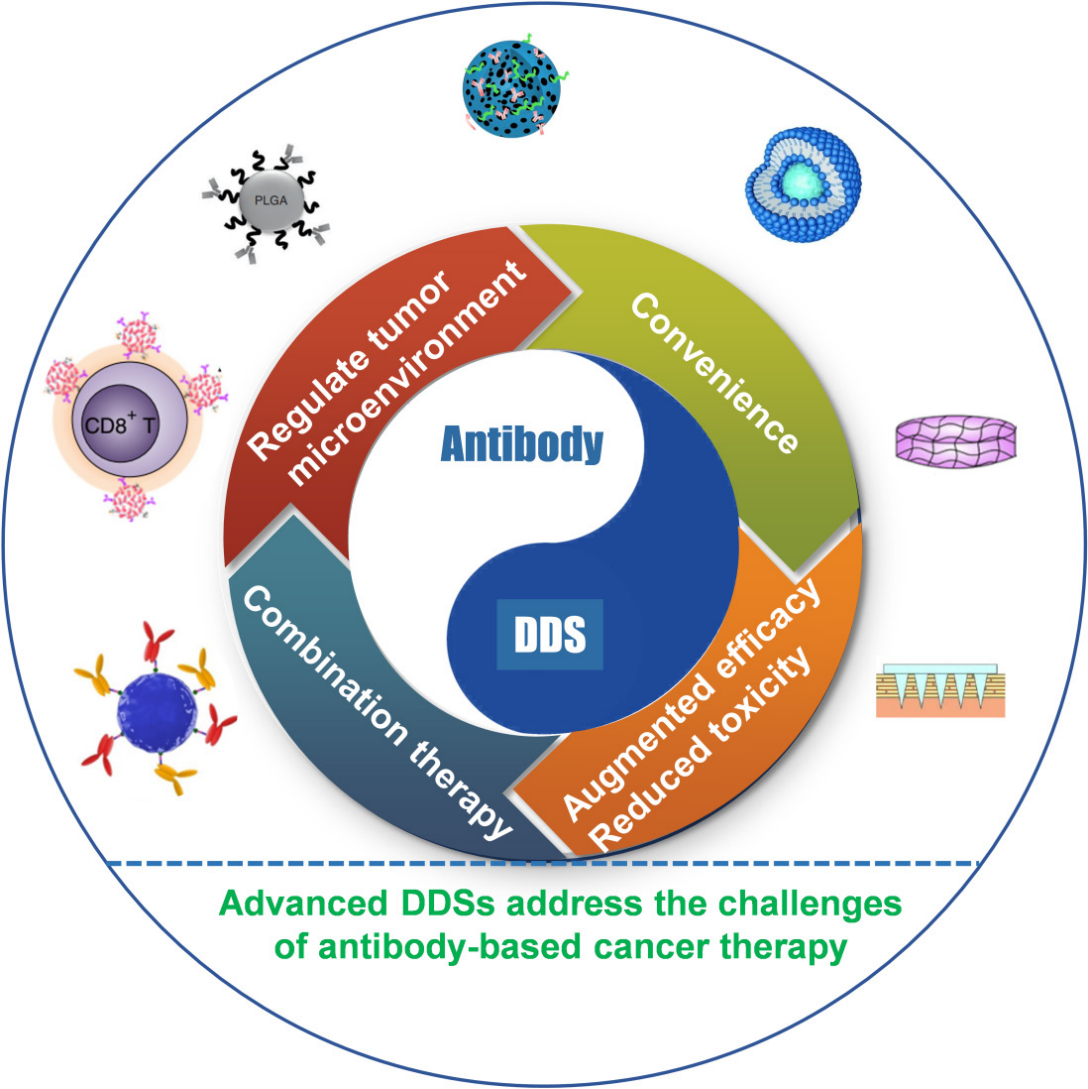
Schematic illustration of various antibody delivery platforms to augment antibody-based therapy efficacy and reduce toxicity, increase convenience, regulate TME, and combination immunotherapy.

Numerous carriers have been developed and applied to deliver various chemotherapeutic drugs, such as liposomes, albumin nanoparticles (NPs), and inorganic materials, among others 56-58. Currently, various carrier-free antibody-based formulations in the market are mainly liquid or lyophilized injections, in which protein stabilizers and osmotic pressure regulators are added to maintain antibody activity and achieve the purpose of injectability. Indeed, the predominant advantage of carriers is to augment antibody-based therapy efficacy and reduce toxicity, increase convenience, regulate TME, and combination immunotherapy (**Figure 2**). Advanced DDSs often exploit one or more of these advantages; in this section, we discuss these aspects, including the recent advancements and latest breakthroughs, highlighting the clinical application prospect of the various delivery systems in antibody-based therapy.

### Encapsulation strategies

**Figure 3:**
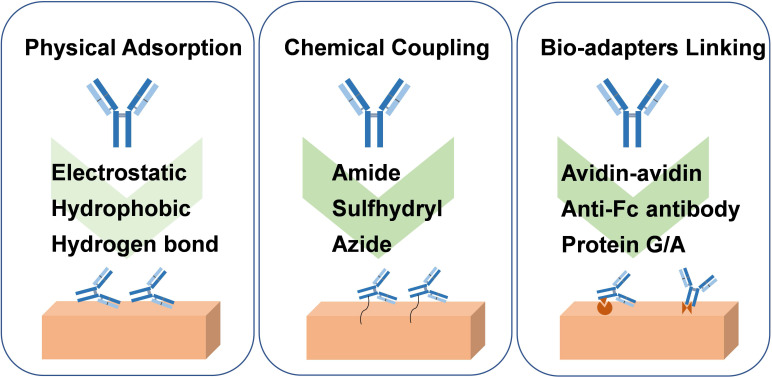
Physical adsorption, covalent binding, and bio-adapters linking strategies for antibody immobilization onto biomaterials.

In DDS, drug carriers can be used for local and systematic delivery of drugs, including liposomes, microspheres, hydrogels, micelles, and mesoporous silica NPs, among others 59, 60. Since antibodies are bioactive biomolecular proteins, they can be encapsulated in liposomes. However, they cannot be blended directly into a material matrix-like small molecule drugs, such as a polymer matrix. On the one hand, it will affect the conformational structure of the protein and may lead to the inactivation of the antibody. On the other hand, as a large molecule, the antibody is challenging to be released from the matrix material. Thus, as seen in **Figure 3**, the main ways of antibody immobilization on the carrier material include physical adsorption and chemical coupling on the material surface, as well strategies of bio-adapter, which has been reviewed by Wang's group 61.

Physical adsorption is widely used for reversible binding of antibodies to the surface of biomaterials, which relies on non-specific interactions between carriers and antibodies, such as electrostatic and hydrophobic interactions, van der Waals forces, and hydrogen bonds. The way of physical adsorption is relatively simple and does not require complex chemical reactions, but the adhesion of physical adsorption is relatively weak, resulting in low load stability. In addition, although the adsorption does not affect the chemical structure of the antibody, it may cause the conformational change of the antibody and then affect the function of the antibody, such as hydrophobic interaction 62. In the case of antibodies loaded on the material surface by physical adsorption, the release of antibodies often depends on the desorption efficiency, which may be affected by temperature, salinity, ionic strength, and pH 63.

For chemical coupling, the use of covalent bonds to attach antibodies to the surface of the biomaterial provides a more potent adhesion force. Antibody molecules include amino, carboxyl, sulfhydryl, and other groups. It can be coupled to the surface of biomaterials through amide reaction and sulfhydryl-related reaction, and an azide group can even be introduced for click reaction, just as the design of the linker for ADC drugs. For amidation reaction, amino acid groups on the surface of antibody (for instance, amino group of lysine, carboxyl group of glutamic acid) can react with a carboxyl group or amino group on the surface of biomaterial and then fix the antibody on the surface of biomaterial to achieve the purpose of loading 6. However, this chemical reaction approach is generally randomly coupled, which may react with the groups in the antigen recognition region, resulting in reducing antigen recognition activity. Moreover, the self-crosslinking reaction between antibodies should be avoided. In this case, enzyme-catalyzed hydrolysis of amide or ester bonds may promote antibody release. In addition, the disulfide bonds in the hinge region and connection region of the antibody can be reduced by reductants to form sulfhydryl groups, which is conducive to Michael's reaction with maleimide or the formation of S-Au bonds with gold 64. The reaction of sulfhydryl with maleimide or gold is mild, but attention should still be paid to the effect on the function of antibodies and appropriate purification measures. The S-Au bond can be broken by irradiation with a femtosecond pulse train to release antibodies. In order not to affect the activity of the antibody, the hydroxyl group on the N-glycans on the antibody with sugar chain structure can be oxidized to the aldehyde group and then react with ammonia or hydrazine on the surface of the carrier 65. Furthermore, it is also an excellent choice to introduce azide group into the specific site of antibody for click reaction, which can better maintain the antibody activity because the conditions of click reaction are also mild 66. Moreover, incorporating the pH-sensitive benzoic-imine bond in the molecular chain of the azide group can facilitate the release of antibodies under the slightly acidic environment of tumor tissue.

Bio-adapters enable stronger antibody attachment than reversible physical adsorption. General methods include coupling antibodies to materials using biotin-binding proteins, Fc-binding proteins, and aptamers. Therein, avidin and streptavidin proteins offer strong binding forces to biotin, which are not easily affected by external conditions, such as pH and temperature 67. In addition, Fc-binding peptides and protein G/A are simpler ways to immobilize antibodies than biotin-binding proteins. Notably, the mild reaction conditions and simple purification process can maintain the activity and stability of the antibody 68. Moreover, after anti-Fc antibody modification on the surface of the biomaterials, it can specifically bind to the Fc fragment of the antibody to realize immobilization. Furthermore, aptamers bind antibodies to biomaterials through complementary base pairing, showing good reproducibility, storability, and low immunogenicity 69. Similar to the chemical coupling method, the antibody loaded in the form of Bio-adapter linking can be released in response to different physiological environments by designing sensitive groups, such as pH, enzyme, and redox, among others.

### Strategies to improve the convenience of medication

The convenience of medication is an important topic. For example, insulin, another protein drug, the routine administration of insulin is subcutaneous injection, which brings some pain and inconvenience to patients. Therefore, researchers try to develop subcutaneous long-term sustained-release preparations and oral dosage forms of insulin. Furthermore, technological platforms for oral delivery of antibodies have also been put on the agenda by scientists 70. Antibody-based drugs are mostly administered intravenously, which brings certain risks, such as infusion-related reactions. Subcutaneous administration is generally more popular with patients than intravenous infusion because it allows self-administration and mobile therapy. At the same time, the long-time administration process brings inconvenience to patients and puts forward higher requirements for medical facilities and equipment. Based on this, Darzalex Faspro^®^ and Herceptin Hylecta® have been developed for subcutaneous administration. It was reported that the tolerance of subcutaneous trastuzumab was similar to that of intravenous trastuzumab, and subcutaneous trastuzumab was non-inferior to intravenous trastuzumab in complete remission rate 41. In order to achieve the dosage, the antibody concentration in such formulation is often considered because the volume injected subcutaneously is often about a few milliliters. More importantly, this formulation is supplemented with hyaluronidase to make antibodies widely distributed in the interstitial space by degrading hyaluronic acid, and eventually, the antibodies enter the bloodstream 41. Thus, combined with the drug carrier research on controlled release of drugs, local administration and long-term sustained release are important ways to improve the convenience of drugs.

#### Local “depots”

**Figure 4:**
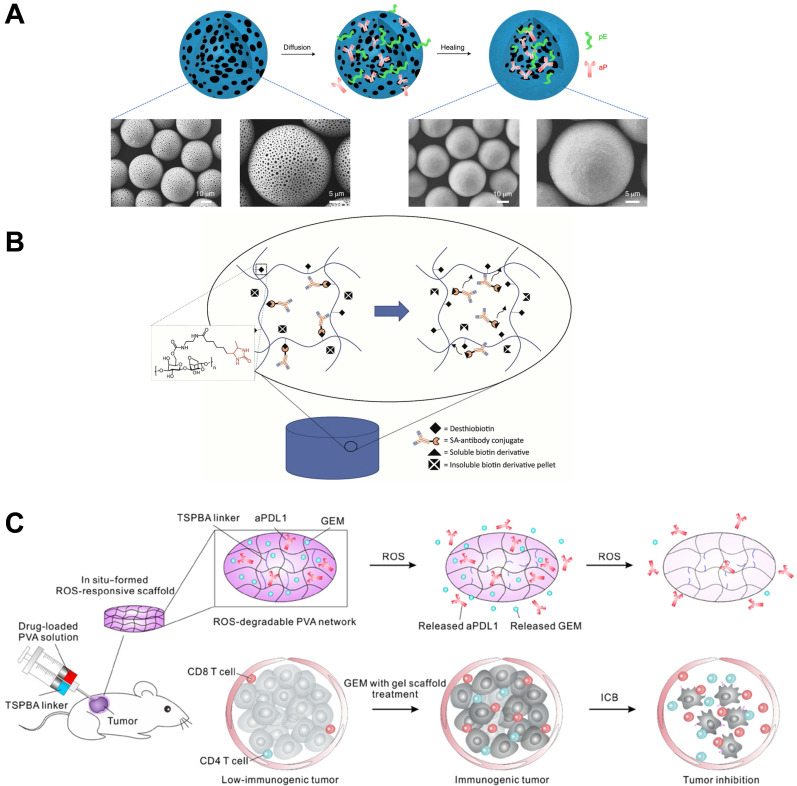
** (A)** Schematic illustration of the preparation of self-healing antibody-loaded microcapsules. Reproduced with permission from Ref. 73, Copyright © 2019 Elsevier. **(B)** Schematic illustration of streptavidin-antibody complexation with agarose-desthiobiotin is disrupted by the dissolution of solid biotin derivative pellets, releasing the antibody conjugate from the hydrogel. Reproduced with permission from Ref. 77, Copyright © 2018 Wiley-VCH GmbH. **(C)** Schematic of combination chemoimmunotherapy using a ROS-degradable hydrogel scaffold to deliver gemcitabine and anti-PD-L1 antibody into the tumor tissue. Reproduced with permission from Ref. 78, Copyright © 2018 American Association for the Advancement of Science.

Long-acting depot formulations have been widely approved for clinical use, and poly(lactic acid) PLA and poly(lactide-*co*-glycolide) PLGA are often used as carriers because of their excellent biocompatibility and degradability. In the depot formulations, small molecule drugs (such as leuprolide acetate, goserelin acetate, doxycycline hyclate, and buprenorphine) and polypeptide drugs (such as somatotropin, lanreotide, exenatide, and pasireotide) are often used as active pharmaceutical ingredients. They are made into polymer-based microspheres, solid implants, *in situ* gels for long-term release within 1 week to 6 months 71. However, as mentioned above, as a macromolecular protein drug, antibodies are generally not mixed in solid polymer matrix like small-molecule drugs because it will affect the conformational stability of the antibodies and cannot be released effectively. Rajagopal and colleagues developed an *in situ*-forming polymer-solvent system for the long-term release of antibody fragments with a molecular weight of 45 kDa 72. The antibody fragment was prepared into spray-dried powders in the delivery system and then dispersed in PLGA-triacetin solution. The designed injectable form exhibited sustained release of antibody fragments for nearly 80 days. However, the preparation of antibody powder is cumbersome by drying, and the use of organic solvents triacetin increases the difficulty of applying this system. To address this aspect, Li and coworkers provided a new strategy to load anti-PD-1-antibody and epitope peptides into closed microcapsules for long-term release 73. In their conception, leukemia-associated epitope peptide and anti-PD-1 antibody were co-encapsulated in PLA microcapsules and then released continuously to recruit activated antigen-presenting cells in the local site, which greatly increased the absorption of epitope peptide by activated APCs and induced T cell modulation by delivering anti-PD-1 antibody to lymph nodes. The immunotherapeutic modality showed superior therapeutic outcomes in leukemia-bearing mice by the potent proliferation and activation of cytotoxic lymphocytes. To prepare peptide and antibody-loaded microcapsules, the porous microspheres were prepared by double emulsion and solvent extraction, then the peptide and antibody were adsorbed in the microspheres by incubation (**Figure 4A**). Finally, the microcapsules were healing at 38 ºC by infrared irradiation. The conditions were mild, which greatly improved the stability of the encapsulated antibody. Due to the gradual degradation of PLA, microcapsules could continuously release antibodies and peptides in nearly 40 days. Inspired by the conception, this strategy is expected to be a candidate for local long-acting controlled-release antibodies.

Hydrogels have rarely been approved for clinical use as a form of drug delivery. However, hydrogels have been widely studied for controlled release drugs because of their excellent biocompatibility, biodegradability, and controllable structure 74, 75. Brandl and colleagues reported degradable thermoresponsive hydrogels by cross-linking of poloxamine derivatives to release antibodies 76. Poloxamine was branched and terminally functionalized by maleimide or furyl groups to prepare the four- and eight-armed macromonomers. The macromonomers aqueous solution was fluid but formed hydrogel by increasing the temperature to 37 °C, due to the Diels-Alder reaction-based covalently cross-linking. The authors stated that the stability of the hydrogel and the release rate of the model antibody bevacizumab could be controlled by adjusting the ratio between four- and eight-armed macromonomers, and the release period could be from 7 days to 115 days. In addition, Wylie and Huynh developed a more sophisticated antibody delivery hydrogel with controlled release of antibody-streptavidin conjugates from the agarose-desthiobiotin group by regulating the dissolution of a small amount of soluble biotin derivatives (**Figure 4B**) 77.

**Figure 5:**
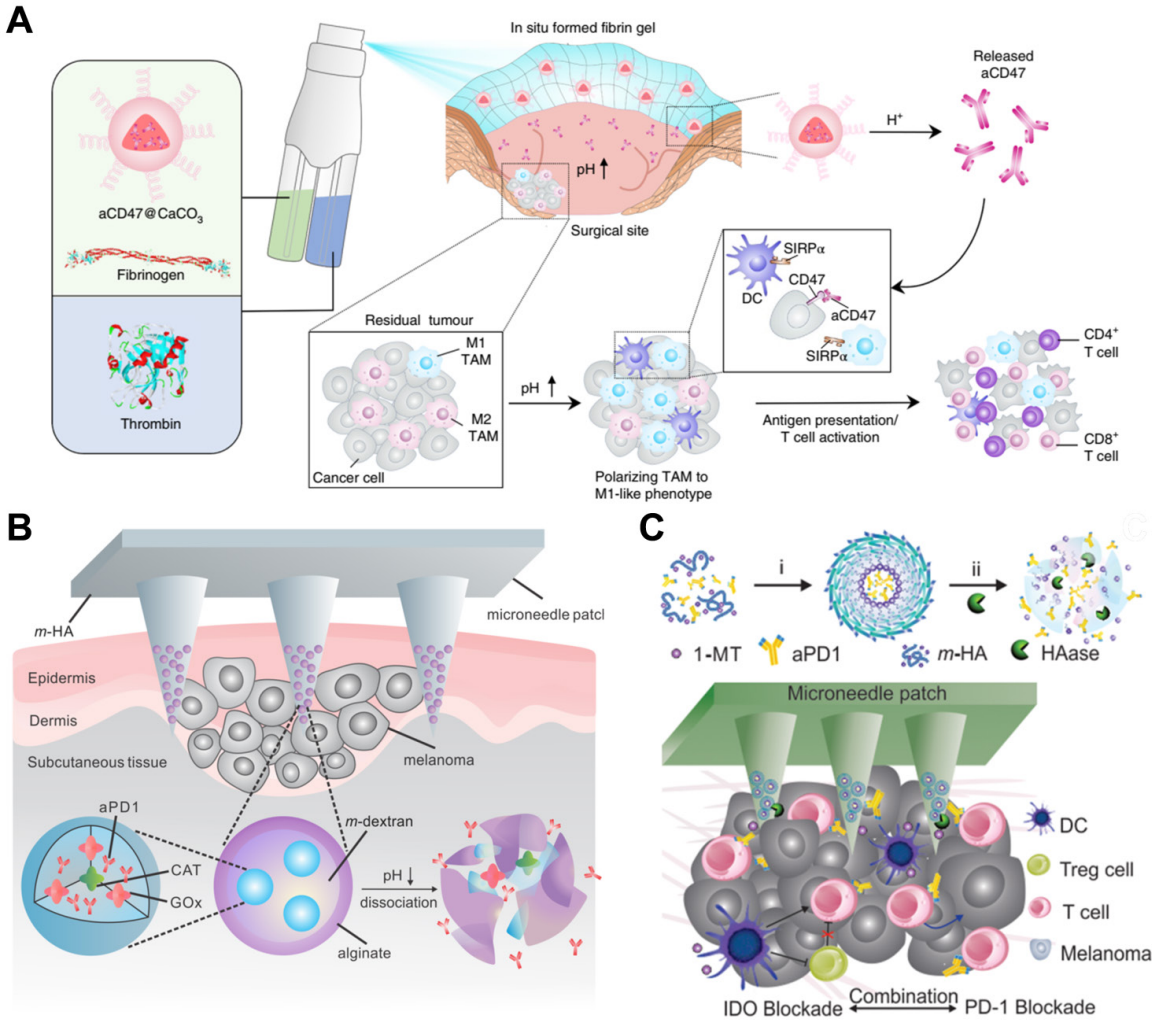
** (A)** Schematic showing the *in situ* sprayed bioresponsive fibrin gel containing aCD47@CaCO3 NPs within the post-surgery tumor bed. Reproduced with permission from Ref. 79, Copyright © 2019 Springer Nature. **(B)** Schematic of the anti-PD-1 antibody delivered by a microneedle patch loaded with physiologically self-dissociated NPs. With GOx/CAT enzymatic system immobilized inside the NPs by double-emulsion method, the enzyme-mediated conversion of blood glucose to gluconic acid promotes the sustained dissociation of NPs, subsequently leading to the release of anti-PD-1 antibody. Reproduced with permission from Ref. 81, Copyright © 2016 American Chemical Society. **(C)** Schematic illustration of encapsulation and release of IDO inhibitor 1-MT and anti-PD-1 antibody from self-assembled NPs. Reproduced with permission from Ref. 82, Copyright © 2016 American Chemical Society.

Competitive affinity release happened to be favorable due to the disruption of protein hydrogel interaction by competing adhesives. Therefore, the release rate of antibodies could be influenced by regulating the concentration and affinity of competitive adhesives. Based on competitive affinity, the hydrogel extended the protein release to 100 to 150 days. These two types of hydrogels provide strategies for long-acting controlled release antibodies. However, the development of such hydrogels needs further experiments *in vivo*. Gu and colleagues developed an *in situ* formed hydrogel with reactive oxygen species (ROS)-responsive characteristics and used for the local release of chemotherapeutic gemcitabine and anti-PD-1 antibody (**Figure 4C**) 78. The drug release experiment showed that the antibody released about 80% in 3 days, which was faster than the gemcitabine completely released in 1 day. As a biodegradable TME-responsive hydrogel which crosslinked poly(vinyl alcohol) with a ROS-labile linker, loaded chemotherapy chemotherapeutic, and ICI inhibitor showed a good combined therapeutic effect in tumor mouse models. Furthermore, the same research group then developed an *in situ* sprayed fibrin gel for post-surgical tumor therapy 79. As shown in **Figure 5A**, the fibrinogen solution containing anti-CD47 antibody-loaded calcium carbonate NPs, and another thrombin solution could quickly mix by the spray set in the post-operative tumor resection cavity, leading to the formation of fibrin gel. Calcium carbonate NPs can gradually dissolve and release the encapsulated antibody to block the “don't eat me” signal in the tumor acidic microenvironment, making tumor cells removed by macrophages. These two kinds of hydrogels designed by Gu's group are not only promising ideas for the treatment of tumors but also very convenient drug formulations. Future evaluations, such as long-term toxicity and dose optimization, are needed for potential clinical translation.

Microneedle patches are a kind of local transdermal drug release formulation with strong penetration and convenient administration ability. Small-molecule and macromolecular drugs can be directly delivered to the dermis through microneedles and have good curative effects on some long-term diseases 80. To develop microneedle patches for use in tumor therapy, Gu and coworkers designed novel biodegradable microneedle patches to treat skin cancer by delivering anti-PD-1 antibodies (**Figure 5B**) 81. The main matrixes of microneedle patches were made by hyaluronic acid, showing excellent biocompatibility and mechanical property, as well as some additives required for photo-crosslinking. In addition, anti-PD-1 antibody and glucose oxidase were encapsulated in pH-sensitive dextran NPs, integrated with hyaluronic acid matrixes. The microneedle patches could penetrate the epidermis and immerse in interstitial fluid, and dextran NPs continuously release anti-PD-1 antibodies by “self-dissociation” in the acidic environment generated after catalyzing glucose. This transcutaneous delivery platform showed robust immune responses to inhibit melanoma growth in mouse models. In another case, Gu's group engineered a synergistic transcutaneous microneedle patches platform to deliver anti-PD-1 antibody and immunosuppressive enzyme indoleamine 2,3-dioxygenase (**Figure 5C**) 82. Microneedle patches could serve as excellent platforms for local long-term release of antibodies, especially for superficial tumors, such as skin cancer and breast cancer. Convenient administration is a major advantage of the microneedle, which also plays a significant role in reducing systemic toxicity and enhancing curative effects. At the same time, it can also be a platform for combined therapy. Nevertheless, the bioavailability of the antibodies in the patches needs further optimization, and the process for making microneedle patches also needs to be more suitable for commercial production.

#### Intravascular “depots”

It is generally believed that most systemic drug delivery is administered intravenously. As mentioned above, among the antibody-based drugs for cancer treatment, only Darzalex Faspro® and Herceptin Hylecta® are modified as subcutaneous injections, and the rest require intravenous administration. There is also a need for improved ease of administration for some intravenously administered antibodies. For instance, long-time infusion and frequent drug delivery times bring inconvenience and pain to patients. From this perspective, the improvement of systematic drug delivery has excellent commercial application prospects. For instance, blinatumomab has been approved to treat adults and children with B-cell ALL, showing remarkable efficacy. However, it usually requires a continuous intravenous infusion of drugs for several weeks, and the medical equipment for infusion is also highly demanding. In addition to the previously described strategy of local sustained antibody release, several promising forms of antibody-carried biomaterials will be discussed next to address the challenge of sustained release of antibodies in the bloodstream.

**Figure 6:**
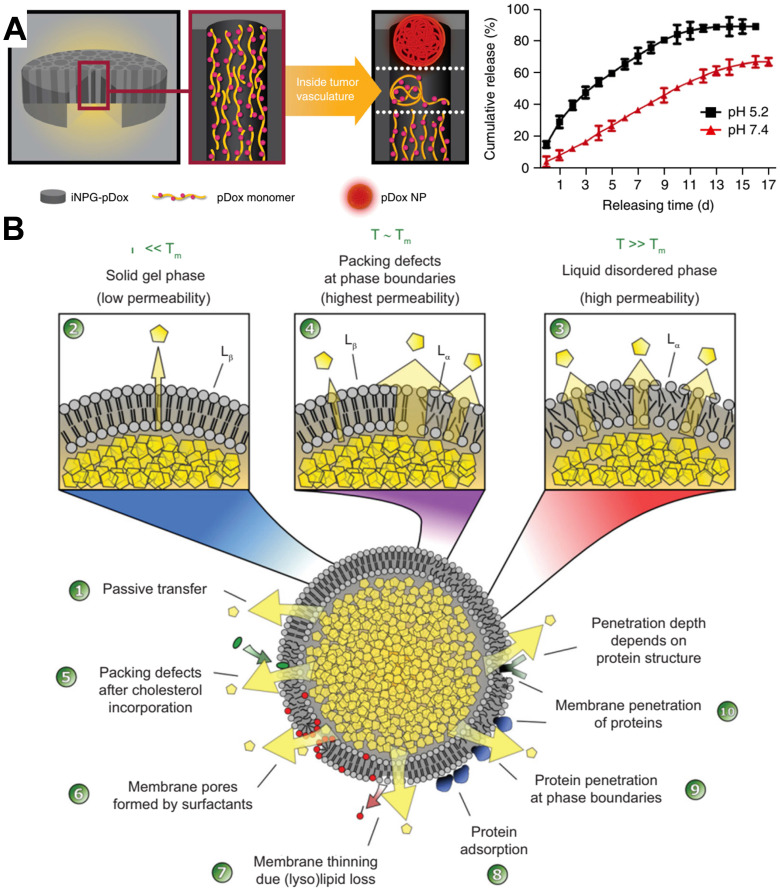
** (A)** Schematic diagram depicting iNPG-pDox composition, pDox prodrug encapsulation, and pDox NP assembly and release from nanopores, and Release of pDox or disassembled Dox from iNPG-pDox at pH 7.4 and pH 5.2 in 10% FBS. Reproduced with permission from Ref. 83, Copyright © 2016 Springer Nature. **(B)** Factors affecting drug release from thermosensitive liposomes. Reproduced with permission from Ref. 85, Copyright © 2020 Elsevier.

Ferrari and colleagues reported an injectable mesoporous silicon particles-based system for various therapeutic applications 83. Silicon microparticles were prepared by complex etching techniques and then modified with amino by conjugating 3-aminopropyltriethoxysilane 84. The mesoporous particles have a diameter of 2.5 µm and a thickness of 700 nm, which are biocompatible and completely biodegradable and can be degraded in saline and completely dissolved in orthosilicic acid within 24 hours. As showed in **Figure 6A**, the discoidal silicon microparticles could be loaded with polymeric drug (pDox), after intravenous injection, the silicon carriers were tightly attached to the tumor microvessels due to the dimensions and the geometry. Then, the loaded macromolecular drugs were released and extravasated into the tumor interstitium through EPR. This intravascular drug delivery macrosystem was expected to enter clinical trials to treat metastatic breast cancer. Although the silicon microparticles are loaded with polymer Dox for chemotherapy in the original design, we believe that through a similar design to load antibodies. It may also become an intravascular antibody “depot” for the continuous release of antibodies.

Liposome-based formulations are the most widely used in clinically approved cancer nanomedicines 14. Almost all liposomes are required to directly deliver drugs into tumor cells after the endocytosis. Nonetheless, several exceptions exist; for instance, ThermoDox is a long-circulating thermosensitive doxorubicin-loaded liposomal supplied by IV infusion. Under the design of scientists, it is expected to release chemotherapeutic drugs intravascularly and in the stroma of tumor tissues when to raise the local temperature above 39 °C in combination with microwave hyperthermia or high-intensity focused ultrasound 14. As depicted in **Figure 6B**, when the liposome temperature is below the melting phase transition temperature (T_m_) (*i.e.,* in the solid gel phase), the drug release in the lipid body is mainly diffusion, and the protein in the blood destroys the integrity of the membrane, resulting in the production of small pores. However, when the temperature is close to or higher than the T_m_, the membrane permeability of liposomes is significantly improved 85. The release rate can be regulated by setting the components of liposomes and designing temperature-sensitive liposomes with different T_m_. In addition, stealth technology can make liposomes prolong the blood circulation time *in vivo* and make the half-life reach even 45 hours 86. Under this assumption, it is possible to make antibodies release continuously in the blood vessel even without external physical heating. Although it is challenging to load protein drugs through liposomes, some new technologies, such as microfluidics, are expected to realize scalable manufacturing from bench to GMP 87. Accordingly, the continuous release of antibodies through intravascular “depots” is expected to solve the challenge of continuous infusion for a long time in treating hematologic tumors and solid tumors.

### Augmented efficacy by modification of antibodies with biomaterials

**Figure 7:**
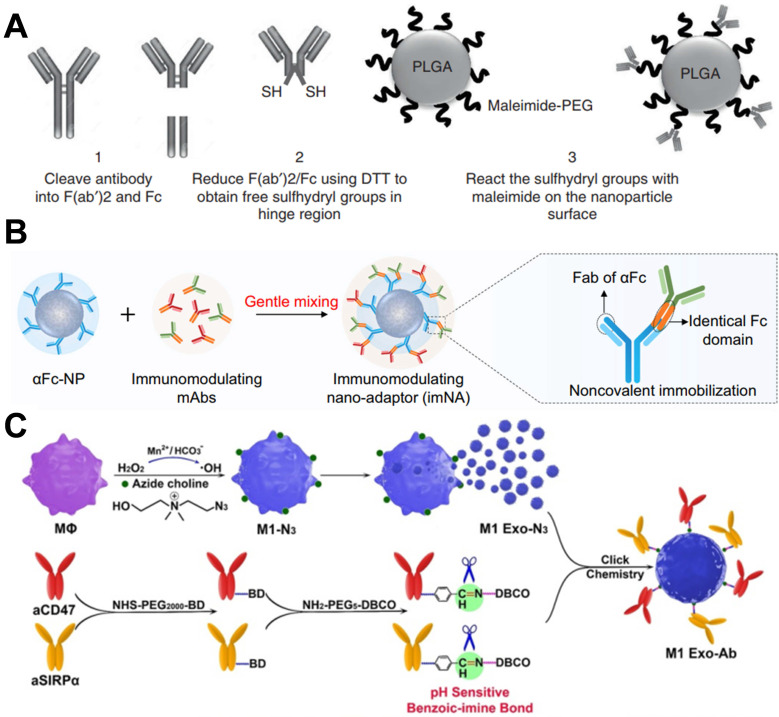
** (A)** Scheme of antibody fragment conjugation to the surface of pre-formulated maleimide-functionalized PEG-PLGA polymeric NPs. Reproduced with permission from Ref. 90, Copyright © 2017 Springer Nature. **(B)** Two types of immunomodulating monoclonal antibodies (mAbs) that target effector cells and tumor cells could be immobilized onto αFc-NP after gentle mixing via Fc recognition. Reproduced with permission from Ref. 91, Copyright © 2021 Springer Nature. **(C)** Scheme of exosome nanobioconjugates (M1 Exo-Ab) composed of M1 macrophage exosomes (M1 Exo) conjugated with immune-stimulatory antibodies linked with pH-sensitive benzoic-imine bonds. Reproduced with permission from Ref. 66, Copyright © 2020 Wiley-VCH GmbH.

In antibody-based drug development, to improve the blood circulation time and increase the retention and accumulation of antibodies in tumor tissues, antibodies-integrated human serum albumin (HSA) fusion protein was developed 88. For example, trastuzumab was conjugated with the HSA domain I fusion protein to maintain high plasma stability while maintaining antibody activity 89. Therefore, using biomaterials to modify antibodies is necessary to enhance the curative effect. In a case, Goldberg and coworkers designed an antibody fragment conjugation to the surface of PLGA NPs, which delivered immunotherapy to endogenous immune cell subsets for improving therapeutic index 90. In their design, antibodies were treated with immunoglobulin-degrading enzyme to cleave IgG molecules below the hinge region, and then reduced by dithiothreitol to obtain sulfhydryl group, which could be chemically coupled with the maleimide group on the surface of PLGA NPs (**Figure 7A**). In addition, the sustained release of payloads in NPs could simulate the paracrine system to reduce tumor immunosuppression. These T cell-targeting nanosystems showed specific and efficient binding for delivery of immunomodulators to endogenous immune cell subsets for delaying tumor growth, as well as breaking the immune tolerance of tumor and increasing the proportion of patients who respond for immunotherapy. In another study, Wang and coworkers designed an immunomodulating nano-adaptor platform to immobilize two different antibodies on one NP 91. As presented in **Figure 7B**, anti-IgG (Fc specific) antibody was coupled on the surface of NPs by oxidation of hydroxyl groups to aldehyde group in the heavy chain CH2 domain, and then condensation with the aminated polystyrene NPs. Based on anti-IgG antibody can specifically recognize and bind Fc fragments of any mAbs through noncovalent interaction, as model mAbs, anti-PD-1 antibody, and anti-PDL-1 antibody were efficiently immobilized on the surface of the NPs. This antibody immobilization platform effectively promoted the interaction between T cells and tumor cells and significantly enhanced the antitumor effect mediated by T cells. Furthermore, this platform could stimulate NK cell- and macrophage-mediated tumor immunotherapy. Meanwhile, the efficacy of such a design is similar to that of multi-specific antibodies, encouraging the clinical translation due to no cumbersome molecular design and genetic engineering.

Furthermore, nanocarriers also offer significant advantages in delivering therapeutic antibodies intracellularly. For some cancers, such as chronic myeloid leukemia (CML), the pathogenic BCR/ABL protein is located inside the cell, and antibodies could not penetrate the cell membrane to achieve the purpose of treatment. Therefore, Feng and colleagues wrapped anti-BCR/ABL antibodies in PLGA NPs and modified the surface with transferrin to improve the ability to target CML cells 92. In this design, anti-BCR/ABL antibodies were delivered to intracellular for BCR/ABL oncoproteins degradation, referring to a good paradigm for intracellular antibody delivery on the way to clinical transformation.

Indeed, the antibodies do not become chemotaxis when they enter the body fluid system, whereas some cells do. Hence, combining the cells with the antibodies would be more potent. In this vein, the platelets act as an essential cellular mediator of thrombosis, tending to migrate and aggregate at damaged blood vessels. Liu et al. developed an anti-PD-L1 engineered platelets-based platform by conjugating anti-PD-L1 antibodies to the surface of platelets *via* chemical linkers 93. Meanwhile, they observed that after photothermal ablation, photodynamic therapy, and high intensity focused ultrasound, transferred platelets should migrate into the tumor tissue after treatment due to the vascular damage and inflammatory microenvironment. With the design, after thermal ablation treatment of tumors, intravenous injection of platelets makes platelets migrate to the tumor area and release antibodies, blocking the effect of PD-L1 on tumor cells and antigen-presenting cells to reduce the growth and metastasis of residual tumors. As a nano membrane-bound vesicle particle produced by cells, exosomes have excellent biocompatibility, stability, long-circulation, and immunogenicity characteristics. Xie and colleagues have co-coupled anti-CD47 and anti-signal regulatory protein alpha (SIRPa) antibodies to M1 macrophage exosomes *via* click chemistry (**Figure 7C**) 66. On the one hand, due to the targeting of anti-CD47 antibodies, the exosome nanobioconjugates could effectively accumulate in tumor tissues and then release antibodies. On the other hand, the M1 macrophage exosomes effectively re-educated M2 to M1 with antitumor effect, while both two antibodies significantly enhanced the phagocytosis of macrophages by blocking the “don't eat me” signal. This strategy not only resulted in a strong synergistic antitumor effect but also reduced the side effects. In addition, Gu and colleagues reported an engineering platelet decorated with anti-PD-1 antibodies, which further conjugated to hematopoietic stem cells for the treatment of leukemia 94. This “cell-combination” strategy skillfully used the homing ability of hematopoietic stem cells and* in situ* activation of platelets, making anti-PD-1 antibodies be released in the bone marrow where the leukemia is located, and effectively promoting the leukemia-specific T cells to control the growth and recurrence of leukemia. Further, Xie and coworkers developed an endogenous CD16-expressing NK cell platform by conjugating trastuzumab and anti-HER2 antibodies to NK cells 95. Similar to CAR-T therapies, this strategy endowed immune cells with tumor-targeting characteristics, offering an excellent curative effect on HER2-expressing tumor treatment and no carcinogenicity towards a potential low-cost cellular-based therapy.

### Combination immunotherapy

Combination therapy using biomaterials loading multiple drugs of different potency simultaneously can improve the therapeutic efficacy. For antibody-based therapies, combination therapy can combine different antibodies or synergistic chemotherapy and phototherapy. In the DDS, loading two or more drugs through biomaterials can avoid the imbalance of drug proportion caused by different metabolic levels of naked drugs and even further optimize the efficacy through sequential controlled release to produce more potent synergistic effects 96.

**Figure 8:**
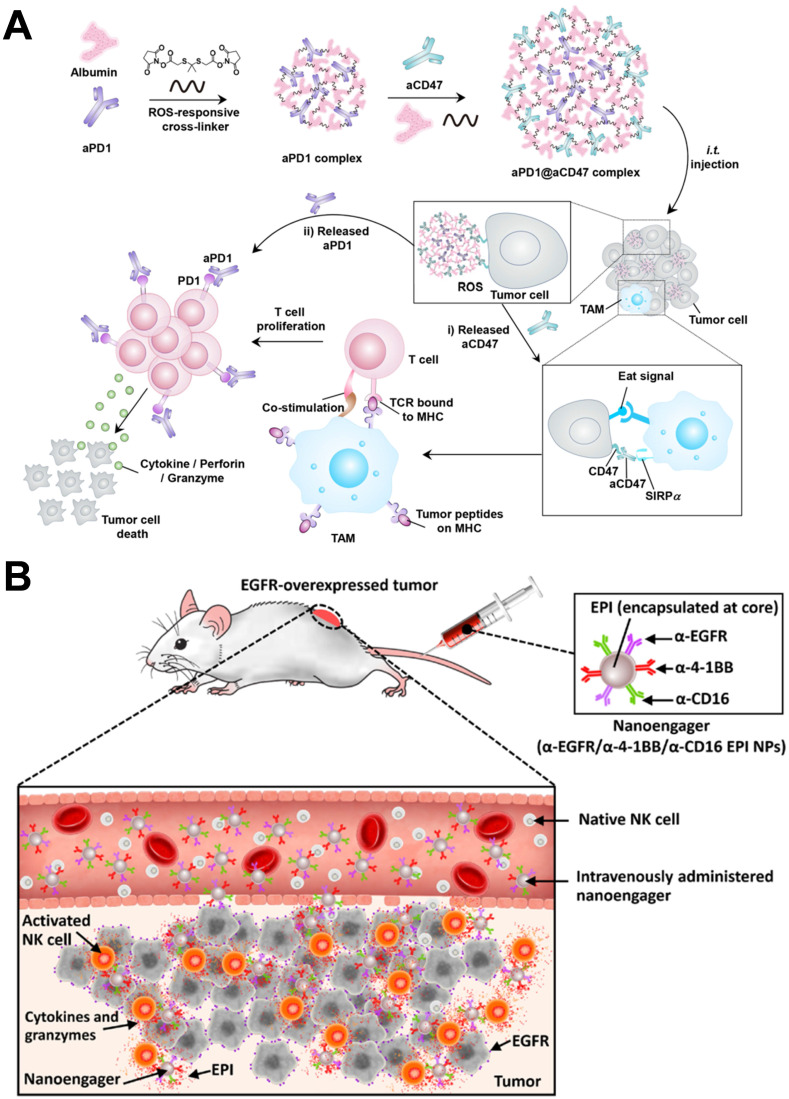
** (A)** Schematic illustration shows synergistic immunotherapy using the ROS-sensitive complexes for controlled sequential release of aCD47 and aPD1 in the tumor microenvironment. Reproduced with permission from Ref. 97, Copyright © 2019 American Chemical Society. **(B)** The cartoon illustrates the mechanism of action of the EGFR-targeted NPbased trispecific NK cell engagers (nano-TriNKEs) (-EGFR/-CD16/-4-1BB NPs) against EGFR-overexpressed cancer after systemic administration. Reproduced with permission from Ref. 99, Copyright © American Association for the Advancement of Science.

Gu and colleagues designed a ROS-responsive albumin complex, in which an anti-PD-1 antibody was wrapped in the core and anti-CD47 antibody in the shell to achieve the sequential release of two antibodies, producing a strong synergistic immunotherapeutic effect (**Figure 8A**) 97. Furthermore, these ROS-degradable complexes could modulate ROS in TME and relieve tumor immunosuppression. Cui et al. engineered to assemble gold nanoclusters using Ce6 molecules as cross-linker, and the nanosystems were conjugated with anti-CD3 antibodies for combined photodynamic and immunotherapy 98. The principle of this design was to internalize the Ce6 and anti-CD3 antibodies-loaded nanoclusters through cytokine-induced killer (CIK) cells and then migrate them to tumor tissue to produce synergistic tumor treatment. At the same time, it could also be used as a diagnostic probe for tumor-targeted imaging. Recently, Wang et al. reported an NP-based chemoimmunotherapy platform, which could target EGFR expressing tumors and enable NK cell-mediated immunotherapy 99. As seen in **Figure 8B**, this platform was referred to as multivalent nanoengagers that chemically coupled with three antibodies: cetuximab, anti-CD16, and anti-4-1BB antibodies. At the same time, the chemotherapy agent epirubicin was also encapsulated in the core of NPs to increase the curative effect. With the help of EGFR targeting, the nanoengagers provided a robust chemoimmunotherapy platform that could recruit and activate NK cells to eliminate tumors and release cytotoxic drugs for tumor chemotherapy.

**Figure 9:**
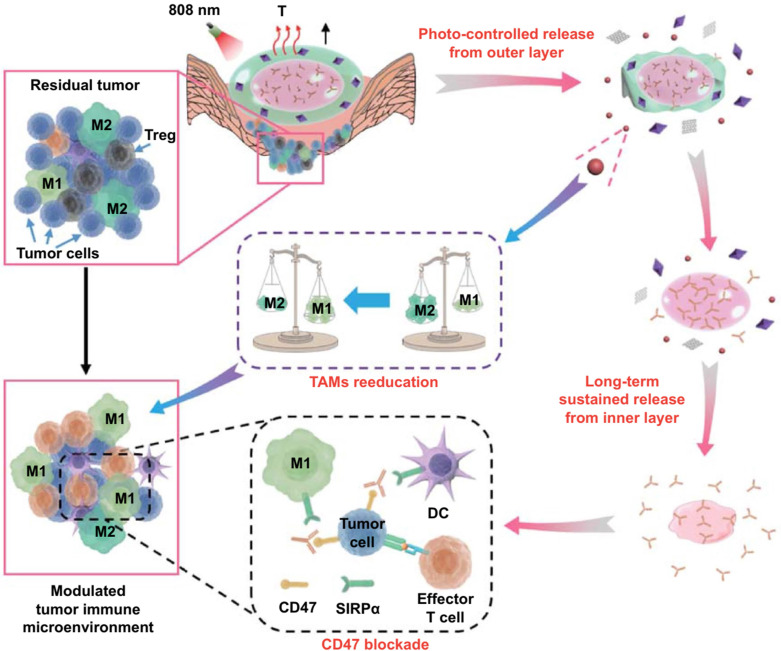
Injectable hierarchical dual lipid gel matrix for local time-programmed sequential delivery of combined cancer immunotherapy. Reproduced with permission from Ref. 100, Copyright © 2021 springer Singapore.

Recently, Luo and coworkers have developed an injectable double‑layer‑gel matrix to time-programmed delivery of multi-kinase inhibitor and antibody to prevent postoperative breast tumor recurrence and metastasis (**Figure 9**) 100. In this system, mixtures with different soybean phosphatidylcholine and glycerol dioleate ratios were used as the inner and outer layers of the lipid gel to regulate the gelation behaviors and drug release profiles. Due to the hierarchical structure, a small molecule multi-kinase inhibitor sorafenib was released in the first layer by irradiating graphene oxide in the gel, causing the temperature to rise slightly, re-educating tumor-associated macrophages. Then, the release of anti-CD47 antibodies was continued in the second layer, reversing immunosuppressive and enhancing the CD47-blockade efficacy. *In vivo* studies showed that this strategy can locally reverse immunosuppression, synergistically block CD47-related immune escape, and effectively prevent tumor recurrence and metastasis.

### Deal with tumor microenvironment

In addition, TME presents a significant effect on the efficacy of immunotherapies by creating variable immune responses. Different types of tumors possess varied microenvironments, including tumor cells, extracellular matrix, peripheral immune cells, and fibroblasts. Notably, the effectiveness of immunomodulatory strategies depends on the specifics of the TME. Immunescore is a new cancer classification method divided into three categories: hot, altered, and cold 12. Among them, hot immune tumors are referred to as immune cells that are highly infiltrated, while cold tumors are significantly lacking immune cells in or around the tumor. In addition, altered ​tumors are divided into ​immunosuppressed and ​excluded, referring to poor immune cell infiltration and no cells infiltration inside the tumor but the accumulation of immune cells at tumor borders. Regulating tumor TME is very important to enhance the efficacy of immunotherapy and can also expand the proportion of immune response patients.

**Figure 10:**
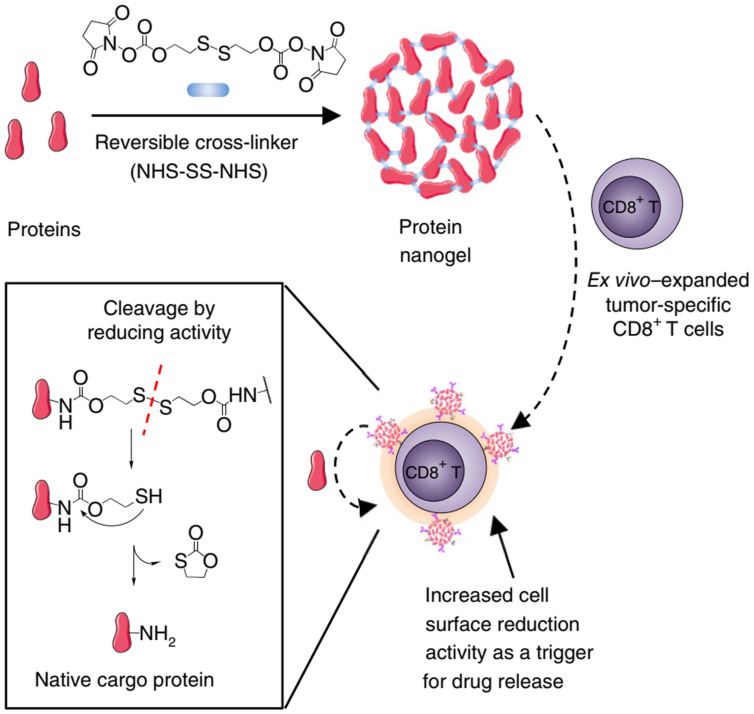
** (A)** Scheme for TCR-signaling-responsive protein nanogels synthesis and for protein release in response to reducing activity in the local microenvironment. Reproduced with permission from Ref. 104, Copyright © 2018 Springer Nature.

Indeed, cellular-based therapy is facing the rapid decline of the vitality and function of transplanted cells. Therefore, adjuvant drugs are often added to enhance the efficacy of cell transfection therapy. However, the delivery of these adjuvant drugs is not precise, as lack of efficacy and dose-associated toxicities limit their clinical use 101. Accordingly, attaching a drug-loaded NP to the adoptive cell surface is an alternative strategy, referred to as “backpack”, in which it's like a backpack full of supplies when you climb a mountain to keep your strength up. In this way, the released payloads augment adoptively transferred T cells' function through autocrine-like behavior and regulate the TME through a paracrine-like manner 102. In 2010, Irvine1 et al. firstly reported a chemical conjugation of liposome-like NPs directly onto T cell membrane, which could continuously stimulate the pseudoautocrine of T cells by releasing adjuvant agent to augment therapeutic potential in animal models 103. CAR-T cell therapy presents a good clinical effect in treating hematological tumors, but it is not ideal in the treatment of solid tumors. In the second generation of T cell “backpack” strategy, IL-15 loaded nanogel backpacks have been linked with adoptively transferred T cells, and the reduction sensitive disulfide bonds in the nanogel was cleaved under reducing conditions on the surface of T cells (**Figure 10**) 104. Fortunately, this strategy has been approved for clinical trials in a variety of solid tumors 105.

Gu and coworkers developed a bioresponsive scaffold that could respond to an acid microenvironment and ROS in the tumor by loading hypomethylation agents, Zebularine, and anti-PD1 antibodies 106. In this aspect, Zebularine could increase tumor immunogenicity and reduce immunosuppression by increasing the expression of tumor-associated antigens so that the anti-PD-1 antibody could produce an anticancer immune response more effectively. Chen and colleagues engineered a magnetic NP conjugated with anti-PD-L1 antibodies, as well as anti-CD3 and anti-CD28 as T-cell activators 107. After intravenous administration, the nanosystem reaches the tumor tissue under the action of magnetic navigation to reduce the side effects. More importantly, it could directly induce T cell activation to repair the immunosuppressive TME. This strategy provided a meaningful reference for changing and enhancing immune responses at specific sites. In addition, to improve the response rates to immune checkpoint blockade therapy, Li's group developed a nanoplatform to combine immunotherapy and photodynamic therapy 108. Crucially, the ROS induced by photosensitizer indocyanine green could promote the infiltration of cytotoxic T lymphocytes and the release of tumor antigens, enhancing the curative effect of PDL-1 blockade. In addition to photodynamic therapy, photothermal effects can also turn “cold” tumors “hot”. Sun et al. encapsulated a photothermal agent and anti-PD-L1 antibody into a gel depot, involved in mild photothermal therapy and immunotherapy 109. When photothermal therapy was evidenced at the injection site, anti-PD-L1 antibodies were released and increased the recruitment of tumor-infiltrating lymphocytes to enhance immunotherapy. In addition to polymer materials, metal-organic frameworks (MOF) can also be used for combined drug loading and synergistic antitumor. Lin et al. reported a nanoscale MOF for co-delivery of toll-like receptor-7 agonist imiquimod and anti-CD47 antibody 110. Due to imiquimod enabled repolarizes M2 macrophages to immunostimulatory M1 macrophages, while anti-CD47 antibodies blocked the “don't eat me” signal for improved phagocytosis. In the colorectal tumor model, this MOF-based nanosystem showed excellent efficacy, resulting in the disappearance of primary and distant tumors.

**Figure 11:**
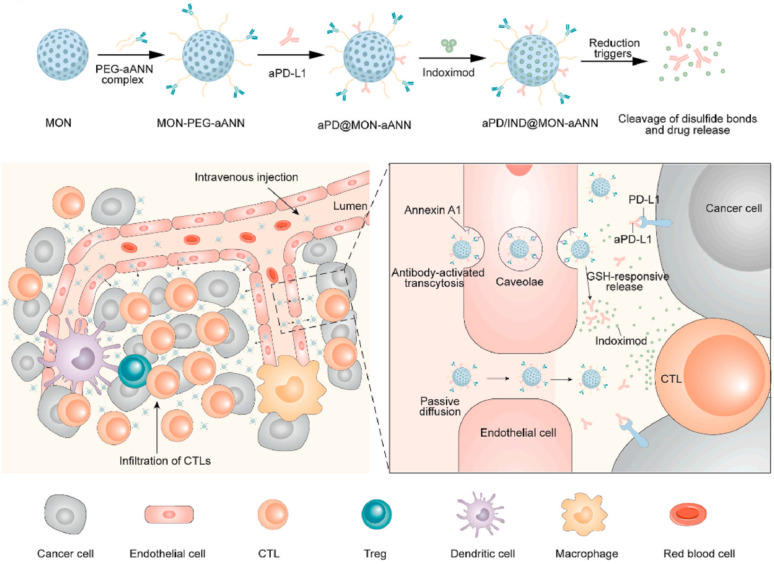
Schematic illustration of Annexin A1 antibody activated trans-endothelial transcytosis to augment nanomedicine extravasation and the efficacy of cancer immunotherapy by synergistic modulation of the tumor microenvironment. Reproduced with permission from Ref. 112, Copyright © 2021 Elsevier.

The enhanced permeability and retention (EPR) effect provides a channel for nanomedicines to enter tumor tissue from blood. Regulating tumor microvessels is also an important measure to enhance the curative effect. Hence, Nie et al. presented a polymer-lipid-peptide NP to deliver the antiplatelet antibody R300 and the chemotherapeutic agent, doxorubicin 111. As an antibody against the platelet von Willebrand factor receptor subunit GPIbα, R300 can deplete platelets, then improve the permeability of blood vessels and increase the accumulation of nanomedicines in tumor tissues. In addition, Annexin A1 antibodies were used to modify the surface of mesoporous organosilica, targeting luminal endothelial cells to activate the caveolae-mediated *trans*-endothelial effect of nanomedicines, leading to enhancing nanomedicine extravasation (**Figure 11**) 112. In this platform, anti-PD-L1 antibody and Indoximod were released from the NPs to reduce TME, resulting in the promotion of the infiltration of cytotoxic T lymphocytes and reversing the immunosuppressive. Under this strategy, nanoplatforms combined immunotherapy involving anti-PD-L1 antibodies and Indoximod can significantly improve the efficacy of solid tumors.

## Outlook and perspective

The development of antibody-based drugs is of primary focus in research, resulting in the development of hundreds of therapeutic mAbs and entry to clinical trials against various diseases. However, in antibody product development, we often pursue better targets to improve the efficacy. In addition, the regulation of antibody characteristics is also the focus of current drug manufacturers, including improving antibody expression and purification efficiency and posttranslational modifications and other regulations 15. In the production of mAb formulations, the influence of protein stabilizer, osmotic pressure regulator, and buffer on antibody activity is often considered, and then the solution or lyophilized dosage form is prepared 113. These formulations in the form of naked antibodies are commonly used for intravenous infusion or subcutaneous injection. However, almost all antitumor mAbs are administered through intravenous infusions. Some exceptions include daratumumab and trastuzumab, developed as both intravenous and subcutaneous administration products. The delivery systems for mAbs have great application potential in reducing toxicity and increasing efficiency, increasing the convenience of drug use, and can bring more substantial product competitiveness for mAbs. Therefore, the development of mAbs should focus not only on antibody engineering but also on DDS.

Indeed, DDSs for the controlled release of antibodies are more challenging than small molecule drugs. However, due to the short half-life of some peptide or protein drugs requiring frequent invasive injections, related controlled-release products have been developed previously. For example, microsphere-based Lupron Depot is used for long-acting releasing leuprolide acetate for up to 6 months, and Nutropin Depot for sustained-release releasing somatropin 51. In addition, non-invasive delivery systems, such as inhalation powder, are also being developed for quick and easy use of drugs 114. Hence, inhalation powder of human insulin and the anti-IL-13 antigen-binding fragment is being developed and applied clinically 51. Even oral insulin was once on the agenda. These efforts have been aimed at developing convenient and effective dosage forms. Generally, the high molecular weight and spatial conformation that affects their bioactivity increase the difficulty of loading and controlling antibodies release. Based on these characteristics of antibodies, physical adsorption or chemical surface grafting is commonly used to load antibodies. In the process of loading and releasing antibodies, maintaining antibody activity and controlling antibody release rate are two key points.

In the local long-acting release system, it is necessary to maintain the antibody activity as far as possible not to be disturbed by the chemical components and enzymes in the tissue. Hence, it is necessary to maintain a suitable environment in the antibody depot, such as avoiding pH changes caused by polymer degradation and preventing the entry of enzymes. Human IgG has a long blood half-life, while some antibody fragments have a very short half-life, making the long-acting release system more attractive to antibody fragments. Generally, after the antibody is released from the tissue, it needs to enter the systemic blood circulation through diffusion, and the bioavailability of each antibody is between 50% - 80% 115. It is unclear whether the bioavailability of the local release system is higher than that of subcutaneous nude antibodies. The bioavailability of subcutaneous administration is affected by protein hydrolysis, antibody aggregation, antibody size, interaction with tissues, and even injection sites 116. In addition, sometimes, the entry of antibodies into the bloodstream is not necessary just to activate the immune function in the local tumor tissue to fight the tumor. Based on these understandings, large porous microspheres, injectable or spray-able hydrogels, and microneedles are expected to achieve long-term controlled release of antibodies and further application. For another, an intravenously sustained release system can be designed when some antibodies cannot be administered subcutaneously because of poor stability or permeability in tissue. In this case, loading antibodies with micro or nanocarriers may not be difficult, and regulating their release behavior in the blood is often the most important. Therefore, using human body temperature or external physical stimulation such as ultrasonic, magnetic field, light, and so on to become alternative methods. Physical stimulation facilitates the release of antibodies in targeted tissues or microvessels. The challenge of long-term release intravascular “depots” is regulating the release rate, such as thermosensitive liposomes, which often have the sudden release in the initial stage and are not conducive to controlling the dose-toxicity of antibodies. On the one hand, the components of these liposomes can be properly screened to meet the requirements of a stable release. On the other hand, external intermittent physical stimulation is also an option, such as ultrasonic and infrared irradiation. In addition, the biocompatibility, biodegradability, and physicochemical properties of such “depots” materials should be strictly required to adapt to the requirements of blood circulation. For ADCs, the payloads are often highly cytotoxic agents with severe side effects, such as peripheral neuropathy, myelosuppression, organ toxicity. On the one hand, the dual warhead ADCs are also being developed by many pharmaceutical enterprises, enhancing the specificity of tumor cells and reducing toxicity 117. On the other hand, developing the local control-released system also helps reduce the toxicity of targeted chemotherapy 118. Moreover, DDSs offer several advantages in the combined treatment of tumors. However, require optimization of the proportion of multiple drugs in the carriers and release of drugs sequentially towards amplified curative effect.

CAR-T therapy is also thought to be antibody-based cell therapy, and the current commercial products are derived from autologous cells, and the indications are blood tumors. In order to maintain the activity of CAR-T cells during proliferation, genetic engineering is often used to edit T cell autocrine to maintain the activity 119. The development of “backpack” can also solve this problem and become a simple and low-cost alternative. For allogeneic CAR-T cells therapy, a gene-editing strategy is also used to reduce host immune rejection. Whether this immune rejection can be shielded by biomaterial strategy may still be an unknown field to be developed. “Backpack” strategy provides active factors for the survival of CAR-T cells in solid TME, which is expected to be used to treat a variety of solid tumors. For the immunosuppressive microenvironment of solid tumors, the tissue penetration of antibodies and immune cells is often limited. Therefore, the development of micro/nano-motors with traction or drag function in drug delivery is also expected to improve the efficacy of immunotherapy 120. In addition, in the development of antibody drugs, pharmaceutical manufacturers often pay more attention to the development of antibodies themselves, such as through antibody engineering and genetic engineering. There exists other alternatives, such as NP grafting of several different antibodies to achieve the efficacy of multi-specific antibodies and “backpack” strategies to boost CAR-T cell activity instead of gene editing. Even, in the later design and development of the antibody itself, by adjusting the properties of the antibody to make it pre-match the existing biomaterial to achieve a better controlled-release effect, rather than allowing the biomaterial to adapt to the existing antibodies.

## Conclusions

As an anti-tumor treatment method developed rapidly in recent years, antibody-based therapy offers both opportunities and challenges. DDS plays an important role in drug efficacy and can produce various benefits to enhance the efficacy of antibody-based therapy. On the one hand, the long-term controlled release can improve the convenience of the antibody-based therapy towards improving the drug's effectiveness and showing good commercialization potential. Strategies such as subcutaneous injection of local long-acting drug delivery formulations, for instance, injectable microspheres, sprayable hydrogels, microneedles, and intravascular “depots” are expected to significantly improve patient medication convenience. On the other hand, the DDS can also reduce the toxicity and increase the efficiency of antibodies in the aspects of sequential controlled release, combined synergy treatment, and regulating the tumor microenvironment. With the rapid development of nanotechnology and researchers' increasing attention to antibody-related dosage forms, more advanced antibody delivery systems will be developed and commercialized and ultimately benefit cancer patients.

## Figures and Tables

**Table 1:** Commercialized immune checkpoint targeting antibodies

Target	mAb	Cancer	Administration	Adverse effects
CTLA-4	Ipilimumab; Yervoy^®^	Melanoma, Colorectal Cancer, RCC, HCC, NSCLC	Intravenous infusion over 30-90 minutes	IRR, IMAR: colitis, hepatitis, endocrinopathies, dermatology, pneumonitis, nephritis, solid organ transplant rejection
PD-1	Nivolumab; Opdivo^®^	Melanoma, colorectal cancer, NSCLC, MPM, RCC, HCC, cHL, SCCHN, ESCC	Intravenous infusion over 30-90 minutes	
	Cemiplimab-rwlc; Libtayo^®^	CSCC, BCC, NSCLC	Intravenous infusion over 30 minutes every 3 weeks	
	Pembrolizumab; Keytruda^®^	Melanoma, stomach, cervical, esophageal, endometrial cancer, NSCLC, SCLC, SCCHN, cHL, MCC, PMBCL, RCC, HCC, TNBC, UC	Intravenous infusion over 30 minutes	
PD-L1	Atezolizumab; Tecentriq^®^	Melanoma, UC, TNBC, HCC, SCLC, NSCLC	Intravenous infusion over 30-60 minutes	
	Avelumab; Bavencio^®^	UC, RCC, MCC	Intravenous infusion over 60 minutes	
	Durvalumab; Imfinzi^®^	UC, NSCLC, SCLC	Intravenous infusion over 60 minutes	

**Table 2:** Commercialized mAbs with Fc-mediated effector mechanisms

Target	mAb	Cancer	Administration	Adverse effects
CD19	Tafasitamab-cxix; Monjuvi®	DLBCL	Intravenous infusion about 1.5-2.5 hours	IRR, myelosuppression, respiratory tract infection, peripheral edema, diarrhea
CD20	Rituximab; Rituxan®	NHL, CLL	In the absence of IRR, intravenous infusion with an increasing infusion rate	IRR, TLS, fever, lymphopenia, chills, infection, asthenia, neutropenia
	Ofatumumab; Arzerra®	CLL		IRR, TLS, neutropenia, leukopenia, pyrexia, cough, diarrhea, anemia, upper respiratory tract infections
	Obinutuzumab; Gazyva®	CLL, Follicular lymphoma		IRR, TLS, hypersensitivity reactions, fatigue, neutropenia, cough, upper respiratory tract infections, neutropenia, thrombocytopenia, musculoskeletal pain
CD38	Daratumumab; Darzalex®	MM	Intravenous infusion using an infusion set fitted with a flow regulator	IRR, upper respiratory infection, neutropenia, thrombocytopenia, diarrhea, constipation, anemia, peripheral sensory neuropathy, fatigue, peripheral edema, nausea, cough, pyrexia, dyspnea, asthenia
	Daratumumab hyaluronidase-fihj; Darzalex Faspro®	MM	Administered subcutaneously, inject 15 mL into the subcutaneous tissue of the abdomen over approximately 3-5 minutes	Hypersensitivity reactions, respiratory tracts infection, constipation, nausea, fatigue, pyrexia, peripheral sensory neuropathy, diarrhea, cough, insomnia, vomiting, back pain, dyspnea
	Isatuximab-irfc; Sarclisa®	MM	Intravenous infusion about 30-60 minutes	IRR, neutropenia, pneumonia, upper respiratory tract infection, diarrhea, anemia, neutropenia, lymphopenia, thrombocytopenia
CD52	Alemtuzumab; Campath®	B-CLL	Intravenous infusion over 2 hours	IRR, cytopenias, cytomegalovirus, infections, nausea, emesis, diarrhea, insomnia
GD2	Dinutuximab; Unituxin®	Neuroblastoma	Intravenous infusion over 10 to 20 hours	IRR, pain, thrombocytopenia, pyrexia, lymphopenia, hypotension, hyponatremia, increased alanine aminotransferase, anemia, vomiting, diarrhea, hypokalemia, capillary leak, syndrome, neutropenia, urticaria, hypoalbuminemia, increased aspartate aminotransferase, and hypocalcemia
EGFR	Necitumumab; Portrazza®	NSCLC	Intravenous infusion over 60 minutes	IRR, cardiopulmonary arrest, hypomagnesemia, venous and arterial thromboembolic, dermatologic toxicities
SLAMF7	Elotuzumab; Empliciti®	MM	Intravenous infusion over 60 minutes using an automated infusion pump	IRR, hepatotoxicity, fatigue, diarrhea, pyrexia, constipation, cough, peripheral neuropathy, nasopharyngitis, upper respiratory tract infection, decreased appetite
CCR4	Mogamulizumab-kpkc Poteligeo®	MF, SS	Intravenous infusion over 60 minutes	IRR, rash, fatigue, diarrhea, musculoskeletal pain, and upper respiratory tract infection
HER2	Trastuzumab; Herceptin®	Breast Cancer, Gastric Cancer	Intravenous infusion over 90 minutes	IRR, headache, neutropenia, diarrhea, fatigue, anemia, stomatitis, weight loss, upper respiratory tract infections, fever, thrombocytopenia, mucosal inflammation, nasopharyngitis, dysgeusia
	Trastuzumab and hyaluronidase-oysk Herceptin Hylecta®	Breast Cancer	Administered subcutaneously over approximately 2-5 minutes	Hypersensitivity, fatigue, arthralgia, diarrhea, injection site reaction, upper respiratory tract infection, rash, myalgia, nausea, headache, edema, flushing, pyrexia, cough
	Margetuximab-cmkb Margenza®	Breast Cancer	Intravenous infusion over 30-120 minutes	IRR, fatigue, nausea, diarrhea, vomiting, constipation, headache, pyrexia, alopecia, abdominal pain, peripheral neuropathy, arthralgia/myalgia, cough, decreased appetite, dyspnea, extremity pain
	Pertuzumab; Perjeta®	Breast Cancer	Intravenous infusion over 30-60 minutes	IRR, hypersensitivity reactions, diarrhea, alopecia, neutropenia, nausea, fatigue, rash, anemia, mucosal inflammation

**Table 3:** Commercialized mAbs for angiogenesis inhibition and receptor signaling blocking pathways

Target	mAb	Indication	Adverse effects
VEGF	Bevacizumab; Avastin®	NSCLC, RCC, HCC, glioblastoma, colorectal cancer, cervical cancer, peritoneal cancer	IRR, epistaxis, headache, hypertension, rhinitis, proteinuria, taste alteration, dry skin, hemorrhage, lacrimation disorder, back pain, exfoliative dermatitis
VEGFR2	Ramucirumab; Cyramza®	Gastric cancer; hepatocellular carcinoma	IRR, hypertension, diarrhea, asthenia, neutropenia, epistaxis, infections, thrombocytopenia, stomatitis, hypoalbuminemia
PDGFRα	Olaratumab; Lartruvo®	Soft tissue sarcoma	IRR, lymphopenia, neutropenia, thrombocytopenia, hyperglycemia, diarrhea, hypophosphatemia
EGFR	Panitumumab; Vectibix®	Colorectal cancer	IRR, dermatologic and soft tissue toxicity, paronychia, fatigue, nausea, and diarrhea
	Cetuximab; Erbitux®	Colorectal cancer, head and neck cancer	IRR, cardiopulmonary arrest, cutaneous adverse reactions, headache, diarrhea, infections

**Table 4:** Commercialized ADC drugs

Target	mAb	Payload	Indication	Adverse effects
HER2	Ado-trastuzumab emtansine; Kadcyla®	DM1	Breast Cancer	IRR, fatigue, nausea, musculoskeletal pain, hemorrhage, thrombocytopenia, headache, increased transaminases, constipation, epistaxis, arthralgia
	Fam-trastuzumab deruxtecan-nxki; Enhertu®	DXd	Breast cancer, Stomach cancer	IRR, neutropenia, left ventricular dysfunction, fatigue, vomiting, alopecia, diarrhea, nausea, hypokalemia, pyrexia
CD20	Ibritumomab tiuxetan; Zevalin®	Yttrium-90	NHL	IRR, peripheral neuropathy, cytopenias, fatigue, nasopharyngitis, nausea, abdominal pain, asthenia, cough, diarrhea, and pyrexia
CD30	Brentuximab; vedotin; Adcetris®	MMAE	cHL, ALCL	IRR, peripheral neuropathy, hematologic toxicities, hepatotoxicity, fatigue, nausea, diarrhea, neutropenia, upper respiratory tract infection, pyrexia, constipation, vomiting, alopecia, decreased weight, abdominal pain, anemia, stomatitis, lymphopenia, mucositis
CD22	Moxetumomab; pasudodox-tdfk; Lumoxiti®	PE38	Hairy cell leukemia	IRR, edema, nausea, fatigue, headache, pyrexia, constipation, anemia, and diarrhea
	Inotuzumab ozogamicin; Besponsa®	Calicheamicin	B-ALL	IRR, thrombocytopenia, neutropenia, infection, anemia, leukopenia, fatigue, hemorrhage, pyrexia, nausea, headache, febrile neutropenia, transaminases increased, abdominal pain, γ-glutamyltransferase increased, hyperbilirubinemia
CD33	Gemtuzumab; ozogamicin; Mylotarg®	Calicheamicin	AML	IRR, hemorrhage, infection, fever, nausea, vomiting, constipation, headache, rash, mucositis, febrile neutropenia, and decreased appetite
CD79β	Polatuzumab; vedotin-piiq; Polivy®	Auristatin	DLBLC	IRR, neutropenia, thrombocytopenia, anemia, peripheral neuropathy, fatigue, diarrhea, pyrexia, decreased appetite, pneumonia
CCR4	Sacituzumab govitecan-hziy; Trodelvy®	Camptothecin	TNBC, urothelial cancer	IRR, neutropenia, nausea, diarrhea, fatigue, alopecia, anemia, vomiting, constipation, decreased appetite, rash, abdominal pain
NECTIN4	Enfortumab vedotin-ejfv Padcev®	Auristatin	Urothelial cancer	IRR, hyperglycemia, hyperglycemia, peripheral neuropathy, ocular disorders, diarrhea
BCMA	Belantamab mafodotin-blmf; Blenrep®	MMAF	MM	IRR, thrombocytopenia, keratopathy, nausea, blurred vision, pyrexia, fatigue

**Table 5:** Commercialized BsAbs and CAR-T cells

Target	mAb	Cancer	Administration	Adverse effects
CD19×CD3	Blinatumomab; Blincyto®	B-ALL	Hospitalization is recommended, continuous intravenous infusion over 24 hours or 48 hours at a constant flow rate using an infusion pump	IRR, infections, pancreatitis, pyrexia, headache, anemia, febrile neutropenia, thrombocytopenia, and neutropenia
CD19	Lisocabtagene maraleucel; Breyanze®	DLBCL	For autologous use only, intravenous infusion	IRR, CRS, fatigue, musculoskeletal pain, nausea, headache, encephalopathy, infections, decreased appetite, diarrhea, hypotension, tachycardia, dizziness, cough, constipation, abdominal pain, vomiting, and edema
	Tisagenlecleucel; Kymriah®	B-ALL, DLBCL	For autologous use only, intravenous infusion	IRR, CRS, infections, fever, diarrhea, nausea, fatigue, hypotension, edema, bleeding episodes, dyspnea, headache
	Brexucabtagene autoleucel; Tecartus®	MCL	For autologous use only, intravenous infusion	CRS, pyrexia, hypotension, encephalopathy, fatigue, tachycardia, arrhythmia, infections, chills, hypoxia, cough, tremor, musculoskeletal pain, headache, nausea, edema, motor dysfunction, constipation, diarrhea, decreased appetite, dyspnea, rash, insomnia, pleural effusion, aphasia
	Axicabtagene ciloleucel; Yescart®	DLBCL, FL	For autologous use only, intravenous infusion	CRS, fever, hypotension, encephalopathy, tachycardia, fatigue, headache, febrile neutropenia, nausea, infections with pathogen unspecified, decreased appetite, chills, diarrhea, tremor, musculoskeletal pain, cough, hypoxia, constipation, vomiting, arrhythmias, dizziness
BCMA	Idecabtagene vicleucel; Abecma®	MM	For autologous use only, intravenous infusion	CRS, infections, fatigue, musculoskeletal pain, hypogammaglobulinemia, diarrhea, nausea, viral infections, encephalopathy, edema, pyrexia, cough, headache, decreased appetite

**Table 6:** Some mAbs-based therapy in late-stage clinical studies for cancer indications

Format	Target	mAb	Indication	Status
Humanized IgG1 (bispecific)	CD20, CD3	Mosunetuzumab	FL	Phase 3
IgG1 (bispecific)	CD20, CD3e	Glofitamab	DLBCL	Phase 3
Humanized IgG1 (bispecific)	HER2, HER2	Zanidatamab	Biliary tract cancer	Pivotal Phase 2
Human IgG4 (bispecific)	BCMA, CD3	REGN5458	MM	Pivotal Phase 2
Humanized IgG4 (bispecific)	GPRC5D, CD3	Talquetamab	MM	Pivotal Phase 2
Humanized IgG4 (bispecific)	BCMA, CD3	Teclistamab	MM	Phase 3 pending
Human IgG4 (bispecific)	CD20, CD3	Odronextamab	NHL	Pivotal Phase 2
Humanized/chimeric IgG1 (bispecific)	PD-L1, CTLA-4	Erfonrilimab	NSCLC	Phase 3
Humanized scFv-scFv, (bispecific)	CD123, CD3	Flotetuzumab	AML	Phase 2
Humanized IgG1 (bispecific)	PD-L1, CTLA-4	KN046	Thymic carcinoma, NSCLC	Phase 3
Human IgG1 (ADC)	CD25	Camidanlumab tesirine	Hodgkin lymphoma	Phase 2
mAb, (ADC)	CEACAM5	Tusamitamab ravtansine	NSCLC	Phase 3
Humanized IgG1 (ADC)	Folate receptor	Mirvetuximab soravtansine	Ovarian cancer, primary peritoneal cancer or fallopian tube cancer	Phase 3
Humanized IgG1 (ADC)	HER2	BAT8001	HER2+ Breast cancer	Phase 3
Humanized IgG1 (ADC)	HER2	Disitamab vedotin	Urothelial carcinoma	Phase 2
Murine IgG1, (radiolabeled)	CD45	Iodine (131I) apamistamab	AML	Phase 3
Murine IgG1	CD276	I-131 Omburtamab	Neuroblastoma metastases	Phase 3
Humanized IgG4	CD47	Magrolimab	Myelodysplastic syndrome	Phase 3
Human IgG1	TIGIT	Tiragolumab	Small cell lung cancer	Phase 3
Humanized IgG4	TIM-3	Sabatolimab	Myelodysplastic syndrome	Phase 3
Human IgG1	PD-L1	Cosibelimab	Squamous cell carcinoma	Phase 3
Murine IgG1	CA125	Oregovomab	Epithelial ovarian cancer	Phase 3
Humanized IgG4	PD-1	HX008	Gastric cancer	Phase 3

## Abbreviations

mAbs: monoclonal antibodies
ADCC: antibody-dependent cell-mediated cytotoxicity
ADCP: antibody-dependent cellular phagocytosis
CDC: complement-dependent cytotoxicity
ADC: antibody-drug conjugate
BsAbs: bispecific antibodies
CRS: cytokine release syndrome
PD-1: programmed death 1
ICI: immune checkpoint inhibitor
ALL: acute lymphoblastic leukemia
AML: acute myeloid leukemia
MDS: myelodysplastic syndrome
DDS: drug delivery system
CAR-T: chimeric antigen receptor T cells
CAR-NK: chimeric antigen receptor natural killer cells
TAA: tumor-associated antigen
TME: tumor microenvironment
CTLA-4: cytotoxic T lymphocyte-associated molecule-4
PD-L1: programmed cell death receptor-1 ligand
TCR: T cell receptor
APC: antigen-presenting cell
RCC: renal cell carcinoma
HCC: hepatocellular carcinoma
NSCLC: non-small cell lung cancer
IMAR: immune-mediated adverse reaction
cHL: classical Hodgkin lymphoma
MCC: Merkel cell carcinoma
UC: urothelial carcinoma
MSI: microsatellite instability
TMB: tumor mutation burden
NK: natural killer cell
EGFR: epidermal growth factor receptor
SLAMF7: signaling lymphocytic activation molecule family member 7
CCR4: CC chemokine receptor type 4
HER2: human epidermal growth factor receptor 2
DLBCL: diffuse large B-cell lymphoma
CLL: chronic lymphocytic leukemia
NHL: non-Hodgkin's lymphoma
SCCHN: squamous cell carcinoma of the head and neck
MPM: malignant pleural mesothelioma
ESCC: esophageal squamous cell carcinoma
CSCC: cutaneous squamous cell carcinoma
BCC: basal cell carcinoma
PMBCL: primary mediastinal large B-cell lymphoma
TNBC: triple-negative breast cancer
IRR: infusion-related reactions
MF: mycosis fugoides
SS: Sézary syndrome
TLS: tumor lysis syndrome
MMAE: monomethyl auristatin E
ALCL: anaplastic large cell lymphoma
PE38: pseudomonas exotoxin 38
MMAF: monomethyl auristatin F
FL: follicular lymphoma
MCL: mantle cell lymphoma
BCMA: B cell maturation antigen
GPRC5D: G protein-coupled receptor class C group 5 member D
TIGIT: T-cell immunoreceptor with Ig and ITIM domains
TIM-3: T-cell immunoglobulin and mucin-domain domain-containing molecule-3
LAG-3: lymphocyte-activation gene 3
HBV: hepatitis B virus
B-CLL: B-cell chronic lymphocytic leukemia
MM: multiple myeloma
VEGF: vascular endothelial growth factor
VEGFR2: vascular endothelial growth factor receptor 2
PDGFR-α: platelet-derived growth factor receptor alpha
EpCAM: epithelial cell adhesion molecule
OS: overall survival
NPs: nanoparticles
PLGA: poly (lactide-*co*-glycolide)
PLA: poly (lactic acid)
ROS: reactive oxygen species
HAS: human serum albumin
CML: chronic myeloid leukemia
SIRPa: anti-signal regulatory protein alpha
MOF: metal-organic frameworks
EPR: enhanced permeability and retention
